# Lipoproteome screening of the Lyme disease agent identifies inhibitors of antibody-mediated complement killing

**DOI:** 10.1073/pnas.2117770119

**Published:** 2022-03-21

**Authors:** Michael J. Pereira, Beau Wager, Ryan J. Garrigues, Eva Gerlach, Joshua D. Quinn, Alexander S. Dowdell, Marcia S. Osburne, Wolfram R. Zückert, Peter Kraiczy, Brandon L. Garcia, John M. Leong

**Affiliations:** ^a^Department of Molecular Biology and Microbiology, Tufts School of Medicine, Tufts University, Boston, MA 02155;; ^b^Department of Microbiology and Immunology, Brody School of Medicine, East Carolina University, Greenville, NC 27858;; ^c^Institute of Medical Microbiology and Infection Control, University Hospital of Frankfurt, Goethe University Frankfurt, D-60596 Frankfurt, Germany;; ^d^Mucosal Inflammation Program, Department of Medicine, University of Colorado Anschutz Medical Campus, Aurora, CO 80045;; ^e^Department of Microbiology, Molecular Genetics, and Immunology, University of Kansas Medical Center, Kansas City, KS 66103

**Keywords:** complement C1, lipoprotein, *Borrelia burgdorferi*, tick-borne disease, serum resistance

## Abstract

Spirochetal pathogens, such as the causative agent of Lyme disease, *Borrelia burgdorferi* sensu lato, encode an abundance of lipoproteins; however, due in part to their evolutionary distance from more well-studied bacteria, such as Proteobacteria and Firmicutes, few spirochetal lipoproteins have assigned functions. Indeed, *B. burgdorferi* devotes almost 8% of its genome to lipoprotein genes and interacts with its environment primarily through the production of at least 80 surface-exposed lipoproteins throughout its tick vector–vertebrate host lifecycle. Several *B. burgdorferi* lipoproteins have been shown to serve roles in cellular adherence or immune evasion, but the functions for most *B. burgdorferi* surface lipoproteins remain unknown. In this study, we developed a *B. burgdorferi* lipoproteome screening platform utilizing intact spirochetes that enables the identification of previously unrecognized host interactions. As spirochetal survival in the bloodstream is essential for dissemination, we targeted our screen to C1, the first component of the classical (antibody-initiated) complement pathway. We identified two high-affinity C1 interactions by the paralogous lipoproteins, ElpB and ElpQ (also termed ErpB and ErpQ, respectively). Using biochemical, microbiological, and biophysical approaches, we demonstrate that ElpB and ElpQ bind the activated forms of the C1 proteases, C1r and C1s, and represent a distinct mechanistic class of C1 inhibitors that protect the spirochete from antibody-mediated complement killing. In addition to identifying a mode of complement inhibition, our study establishes a lipoproteome screening methodology as a discovery platform for identifying direct host–pathogen interactions that are central to the pathogenesis of spirochetes, such as the Lyme disease agent.

## Significance

Spirochetal pathogens encode an abundance of lipoproteins that can provide a critical interface with the host environment. *Borrelia burgdorferi*, the model species for spirochetal biology, must survive an enzootic life cycle defined by fluctuations between vector (tick) and vertebrate host. While *B. burgdorferi* expresses over 80 surface lipoproteins—many of which likely contribute to host survival—the *B. burgdorferi* lipoproteome is poorly characterized. Here, we generated a platform to rapidly identify targets of *B. burgdorferi* surface lipoproteins and identified two paralogs that confer resistance to antibody-initiated complement killing that may promote survival in immunocompetent hosts. This work expands our understanding of complement evasion mechanisms and points toward a discovery approach for identifying host–pathogen interactions central to spirochete pathogenesis.

The spirochete *Borrelia burgdorferi* sensu lato is the etiological agent of a diverse set of symptoms collectively referred to as Lyme disease, which is estimated to infect over 476,000 people annually in the United States (1). *B. burgdorferi* is transmitted to humans and other reservoir hosts—primarily small mammals and birds—via the bite of a nymphal or adult-stage infected hard tick (*Ixodes scapularis*). Upon tick feeding, bacteria are exposed to host blood in the tick midgut and then migrate to the salivary gland to be injected into the host dermis, where they establish a local spreading skin infection reflected in a characteristic expanding rash, erythema migrans (2, 3). The spirochetes then disseminate via the circulatory and lymphatic systems to colonize other sites, such as joints, heart, nervous tissue, and distant skin (4). Spirochetes can then be acquired by other feeding ticks, including larval-stage ticks (5). As transovarial spread of *B. burgdorferi* does not occur in ticks, this feeding step is critical for intergenerational spirochetal transmission and retention of the bacterium in the tick population.

The ability of the spirochete to spread within the vertebrate host is reflected in its ability to cause multisystemic human disease, including arthritis, carditis, neuroborreliosis, and the formation of multiple erythema migrans lesions (6). The interaction of the Lyme disease spirochete with the host extracellular environment promotes its dissemination and persistence and is mediated, in part, by its surface lipoproteome. Spirochetal pathogens encode an abundance of lipoproteins, some of which are located on the bacterial surface (7–9), and in fact most of ∼125 *B. burgdorferi* lipoproteins are surface-localized (10, 11). Many of these lipoproteins recognize identical or related host targets and interact with more than one host ligand (12). For example, at least 11 *B. burgdorferi* lipoproteins recognize host glycosaminoglycans (7), and nearly a dozen more interact directly with components of the innate immune system known as the complement cascade (13, 14). Understanding the interface between the complex *B. burgdorferi* surface lipoproteome and host macromolecules is fundamental to improving disease treatment and pursuing novel vaccine targets. However, due in part to their evolutionary distance from the better-studied bacteria, such as Proteobacteria and Firmicutes, relatively few *B. burgdorferi* lipoproteins have assigned functions.

For both survival during exposure to the bloodmeal in the tick midgut and dissemination of the spirochete throughout the vertebrate host, protection against host defenses is essential. The complement system is the most immediate threat to survival that pathogens must contend with in the blood. This system is composed of a set of soluble and membrane-associated proteins that interact and activate a multistep proteolytic cascade upon detection of microbial surfaces, ultimately forming complexes that can damage microbial membrane integrity, recruit immune cells, and enhance phagocytosis (15–18). The three canonical pathways of complement system activation are each triggered by the recognition of molecular patterns on pathogenic surfaces. The lectin pathway proceeds by the recruitment of serine proteases (MASPs) to mannose-binding lectin bound to the microbial surface by recognition of mannose or related sugars. The alternative pathway is triggered when complement factor C3 undergoes spontaneous cleavage in proximity of a microbial surface; it also serves as the central amplification loop of the complement cascade. The classical pathway (CP) typically initiates through the binding of host C1 to IgG or IgM complexes on the bacterial surface, although pathogen- or damage-associated molecular patterns can also trigger this pathway. All three pathways result in the formation of enzymatic complexes that trigger the release of proinflammatory peptides, the opsonization of the microbe, and the formation of a membrane attack complex (MAC) that lyses the pathogen.

To promote survival during tick feeding and spread within the vertebrate host, *B. burgdorferi* encodes surface lipoproteins that inhibit key steps of complement activation (13, 14, 19). *B. burgdorferi* OspC (outer surface protein C), a lipoprotein essential to the spirochete life cycle, binds to C4b to inhibit *B. burgdorferi* bloodstream clearance (20). In addition, *B. burgdorferi* produces three distinct classes of factor H-binding proteins, termed complement regulator-acquiring surface proteins (CRASPs), including CspA (CRASP-1), CspZ (CRASP-2), and ErpP/ErpC/ErpA (CRASP-3/CRASP-4/CRASP-5) (21–30). Each of these proteins binds factor H, the major negative host regulator of the central amplification loop of the complement cascade and protects the bacterial surface from C3 deposition (31). The timing of expression varies among CRASPs, and CspA is specifically required for tick-to-host spirochete transmission, whereas CspZ mediates dissemination through the bloodstream and into distal tissues (32, 33).

Among known borrelial complement evasion proteins, *B. burgdorferi* BBK32 is unique in its ability to bind the complement C1 complex (34, 35). As the sole activator of the CP, C1 is comprised of the scaffold protein C1q and a heterotetramer of the serine proteases C1r and C1s (i.e., C1qC1r_2_C1s_2_). C1q binding to the Fc region of an engaged antibody activates C1r to cleave C1s, which in turn cleaves complement components C2 and C4, leading to downstream C3 and C5 activation. BBK32 binds the C1 complex by recognizing C1r, blocking C1r proteolytic activity. When ectopically produced in a noninfectious, high-passage, otherwise serum-sensitive *B. burgdorferi* strain, BBK32 confers serum resistance (34). However, in an infectious strain background (i.e., strain B31), a Δ*bbk32* mutant remains resistant to CP-mediated complement killing (34), suggesting that additional borrelial factors protect the spirochete from complement activation through this pathway.

*B. burgdorferi* carries as many as 21 endogenous plasmids, many of which are not stably maintained during in vitro culture, thus complicating genetic approaches to the identification of novel virulence factors (36). Nevertheless, a transposon library of *B. burgdorferi* has previously proved useful for genome-wide screens to identify many virulence factors (37). Unfortunately, functional redundancy of lipoproteins may limit its utility in exploring the genome for host interactions. Alternatively, gain-of-function studies have allowed researchers to detect the acquisition of new virulence-associated functions, such as complement resistance or cell attachment (22, 34, 38, 39). This is accomplished through ectopic lipoprotein production in a high-passage strain that, due to stochastic plasmid loss, lacks many virulence-associated functions and is noninfectious. To comprehensively identify *B. burgdorferi* lipoproteins located on the outer surface of the spirochete, Dowdell et al. (10), using a strong constitutive promoter, ectopically produced epitope-tagged versions of all 127 putative lipoproteins encoded by *B. burgdorferi* strain B31 in the high-passage strain B31-e2, finding that more than 80 are detected on the outer surface.

In this study, we used this library of B31-e2 clones to establish a surface lipoproteome screening methodology. Based on the serum-resistance phenotype of a *bbk32*-deficient mutant described above and the observation that the complement evasion system of Lyme disease spirochetes has evolved to be functionally overlapping, we targeted our lipoproteome screen toward the human C1 complex. Erp proteins, whose genes share a high degree of homology in their promoter regions (23, 40–42; for review, see ref. 43), are comprised of three families, including the Elp family, based on their mature protein sequences (41, 44). We found that two Elp family members, ElpB and ElpQ (formerly termed ErpB and ErpQ, respectively), bind C1 with high affinity and block its activity through inhibition of the C1s protease subcomponent. Furthermore, we show that ElpB and ElpQ promote resistance to antibody-dependent complement killing. The discovery of a unique role for ElpB and ElpQ in evading complement provides a validation of our lipoproteome screening methodology, which may be leveraged again in future studies to better understand the host–pathogen interface of the most prominent vector-borne pathogen in North America.

## Results

### Screening the *B. burgdorferi* Surface Lipoproteome Identifies High-Affinity Interactions between ElpB and ElpQ with Human Complement Component C1.

Utilizing a previously described lipoproteome library, we developed a whole-cell binding assay to screen 80 strains of *B. burgdorferi* B31-e2 that each ectopically overproduce a single distinct C-terminally His-tagged, surface-localized lipoprotein from the *B. burgdorferi* lipoproteome (10) for the ability to adhere to candidate ligands. As nonadherent controls, we included the parental strain B31-e2, as well as a strain that overproduces the lipoprotein BB0460, which was reported to be largely periplasmic (10). To validate our approach, we first screened the library for strains that bind to human fibronectin. As expected, the two strains that bound fibronectin most strongly overexpressed the *B. burgdorferi* outer surface lipoproteins BBK32 and RevA, each of which have been shown to bind human fibronectin (45–49) (*SI Appendix*, Fig. S1 and Table S1).

**Fig. 1.**
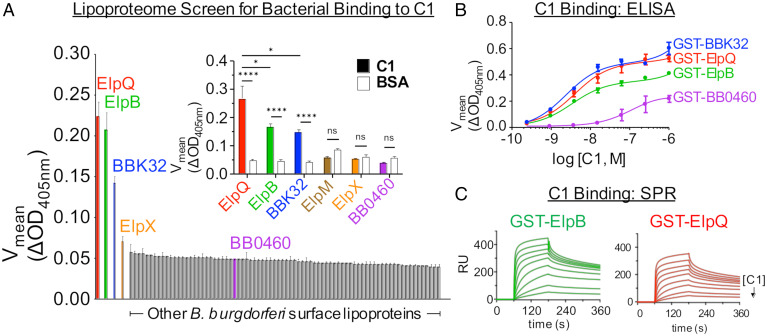
Screening the *B. burgdorferi* surface lipoproteome identifies high-affinity interactions between ElpB and ElpQ with human C1. (*A*) The 1 × 10^6^ strain B31-e2 producing one of 80 *B. burgdorferi* surface lipoproteins (10) (*SI Appendix*, Table S1), as well as a lipoprotein (BB0460) that was reported to be largely periplasmic (10) to serve as a negative control, were applied to microtiter wells coated with human C1 complex in duplicate. After washing, bound bacteria were quantitated by the change in OD_405nm_ over time by ELISA using an anti-*B. burgdorferi* antibody (*Materials and Methods*). The clones are sorted in order of binding signal with raw values shown for each clone in *SI Appendix*, Table S1. Error bars indicate SEM. (*Inset*) Binding of clones producing the indicated *B. burgdorferi* Elp protein, along with a positive control (BBK32) or a negative control (BB0460), to immobilized human C1 complex or BSA was quantitated as described above. Error bars indicate SEM. ****P < 0.0001; *P < 0.05; ns, not significant using Student’s *t* test to compare mean values. (*B*) Binding of the indicated GST-fusion proteins to wells coated with the indicated concentration of human C1 complex was quantitated. The experiment was performed six times (GST-ElpB) or nine times (GST-BBK32 and GST-ElpQ) at each concentration and error bars indicate SEM. Affinity analysis was performed with Prism GraphPad software, using a nonlinear regression analysis. (*C*) The ability for GST-ElpB (*Left*) GST-ElpQ (*Right*) to bind human C1 complex was evaluated by SPR. A twofold dilution series (0.6 to 150 nM) of C1 complex was injected over GST-ElpB and GST-ElpQ biosensors and steady-state affinity analysis was carried out with T200 Evaluation Software. Each SPR experiment was performed in triplicate. Equilibrium dissociation constants (*K*_D_) calculated from ELISA-type and SPR binding assays are shown in Table 1.

To identify surface lipoproteins that target the CP, we screened the library for binding to purified, immobilized, human C1 complex. In addition to binding fibronectin and dermatan sulfate, BBK32 binds C1 (34, 35) and, as expected, spirochetes overexpressing BBK32 bound specifically to C1 in our screen (Fig. 1*A*, blue). Interestingly, strains overexpressing lipoproteins ErpB or ErpQ (referred to in Fig. 1 as “ElpB” and “ElpQ,” respectively, for reasons described below) also bound strongly to C1, exhibiting a relative signal higher than that of the BBK32-expressing strain (Fig. 1*A*).

ErpB and ErpQ are members of the *B. burgdorferi* OspEF-related protein family (Erps), characterized by Marconi, Stevenson, and others (42, 43, 50). The genes encoding those Erp proteins were identified based on the similarity of their promoter sequences, and most of these genes are located on circular plasmid 32 (cp32) DNA elements (23). The analysis of the mature protein sequences indicates that Erp proteins fall into three families: OspE, OspF, and OspE/F-like leader peptides (Elp) (41, 44). Most OspE family members, including the above mentioned ErpP/ErpC/ErpA (CRASP-3/CRASP-4/CRASP-5), bind to the complement regulators factor H or factor H-related proteins (14). OspF and six other Erp proteins have been shown to bind heparan sulfate (38). Finally, Elp family proteins, defined by homology of their OspE/F-like leader peptides (41), which heretofore have had no known shared function, include ErpB and ErpQ. To distinguish ErpB and ErpQ from other Erp proteins based on both homology and apparent function, herein we refer to them as ElpB, ElpQ, and other proteins that share amino acid homology, as Elp rather than Erp proteins.

The genome of *B. burgdorferi* strain B31, the parental strain of B31-e2, encodes not only ElpB and ElpQ, but also ElpM, ElpO, and ElpX (42, 43, 50) (*SI Appendix*, Table S4). Despite being encoded on separate cp32 plasmids, *elpB* and *elpO* are identical at the amino acid sequence level, and for simplicity, ElpO will be referred to as ElpB hereafter. In strain B31, the Elp proteins (i.e., ElpB, ElpM, ElpQ, and ElpX) are 44 to 59% identical and 59 to 76% similar and exhibit their highest identity in the N-terminal and C-terminal protein regions (*SI Appendix*, Fig. S2 and Table S4). Strain B31-e2, a high-passage derivative of strain B31, retains only three cp32 plasmids and does not carry *elpQ*, *elpM*, *elpO*, or *elpX* (51). However, this strain does encode *elpB*; thus, our finding here that strain B31-e2 does not bind to immobilized C1 absent ectopic production of ElpB or ElpQ indicates that the endogenous level of ElpB production and surface localization in this strain is insufficient to promote spirochetal binding in this assay.

To confirm the results of our screen, and because little is known about the function of Elp proteins, we individually tested strains producing each Elp in the ELISA-based spirochete binding assay against the C1 complex, including bovine serum albumin (BSA) as a negative control (Fig. 1 *A*, *Inset*). Spirochetes expressing BBK32 (a C1-binding protein) and BB0460 (a lipoprotein previously suggested to be largely periplasmic) (10), were used as positive and negative controls, respectively. Strains producing ElpB, ElpQ, or BBK32 all exhibited statistically significant binding to C1 relative to BSA, whereas ElpM, ElpX, and BB0460 did not (Fig. 1 *A*, *Inset*).

To further investigate the ability of ElpB and ElpQ to directly bind to human C1, we purified recombinant GST-tagged fusion proteins (GST-ElpB and GST-ElpQ). Consistent with data obtained from the spirochete binding assay (Fig. 1*A*), GST-ElpB and GST-ElpQ bound with high affinity to immobilized C1 in an enzyme-linked immunosorbent assay (ELISA-type) binding assay, exhibiting apparent equilibrium dissociation constants (*K*_D_) of 3.4 nM and 3.8 nM, respectively (Fig. 1*B* and Table 1). To gain insight into the interaction of ElpB and ElpQ with soluble C1, we used surface plasmon resonance (SPR) whereby GST-ElpB and GST-ElpQ were immobilized on SPR sensor chips. When C1 was used as an analyte, strong C1-binding was observed, with GST-ElpB and GST-ElpQ exhibiting steady-state calculated *K*_D_ values of 5.6 and 11 nM, respectively (Fig. 1*C* and Table 1). Together, these data confirm that ElpB and ElpQ individually promote spirochete binding to human C1 via direct interaction with this molecule.

**Table 1.** ELISA-type and SPR binding assays

GST-fusion protein	Complement protein	ELISA *K*_D_ (nM)*	SPR *K*_D_ (nM)^†^
GST-ElpB	C1	3.4 ± 0.4	5.6 ± 1.5
	C1r enzyme	41 ± 4.3	100 ± 27
	C1s enzyme	6.7 ± 0.7	3.9 ± 0.48
	C1r proenzyme	—	NB
	C1s proenzyme	—	270 ± 55
GST-ElpQ	C1	3.8 ± 1.2	11 ± 2.0
	C1r enzyme	11 ± 1.9	97 ± 35
	C1s enzyme	4.7 ± 1.0	4.5 ± 1.0
	C1r proenzyme	—	NB
	C1s proenzyme	—	170 ± 73

NB, no detectable binding.

^*^K_D_ determined by quantitative ELISA.

^†^*K*_D_ determined by SPR.

### ElpB and ElpQ Selectively Bind the Activated Forms of C1r and C1s.

The C1 complex is composed of C1q and a heterotetramer of C1r and C1s (i.e., C1r_2_C1s_2_) (*SI Appendix*, Fig. S3*A*). C1q is a nonenzymatic component and functions in pattern recognition, while C1r and C1s are serine proteases that catalyze the initial proteolytic reactions of the CP. To clarify whether ElpB and ElpQ bind to C1 by interacting with individual subcomponents, we carried out an ELISA-type binding assay using purified immobilized C1q and activated forms of C1r and C1s (i.e., C1r enzyme and C1s enzyme). Relative to the negative control GST-BB0460, no significant interaction was detected for either GST-ElpB or GST-ElpQ with human C1q, (*SI Appendix*, Fig. S3*B*). In contrast, each protein bound with high affinity to C1r enzyme (*K*_D_ of GST-ElpB/C1r = 41 nM; GST-ElpQ/C1r = 11 nM) as well as to C1s enzyme (*K*_D_ of GST-ElpB/C1s = 6.7 nM; GST-ElpQ/C1s = 4.7 nM) (Table 1 and *SI Appendix*, Fig. S3 *C* and *D*).

To examine the function of ElpB and ElpQ in binding C1 subunits when produced by *B. burgdorferi*, we first assessed the relative amounts of ElpB and ElpQ in bacterial lysates by conventional immunoblotting, detecting the His tag on the ectopically produced lipoprotein. ElpB and ElpQ migrated with their predicted apparent molecular masses (61 kDa and 55 kDa, respectively). Full-length ElpQ was produced at vastly higher levels than ElpB, and the presence of a prominent lower molecular weight ElpB band—presumably a stable degradation product—suggested that ElpB, but not ElpQ was subjected to proteolytic cleavage (*SI Appendix*, Fig. S4). Pronase treatment was then used to assess the surface localization of each protein, as previously described (10). As expected, ElpB and ElpQ were predominantly expressed on the spirochetal surface (*SI Appendix*, Fig. S4). *B. burgdorferi* B31-e2 producing BBK32 or BB0460, analyzed in parallel, were exclusively or predominantly, respectively, localized on the bacterial surface.

**Fig. 2.**
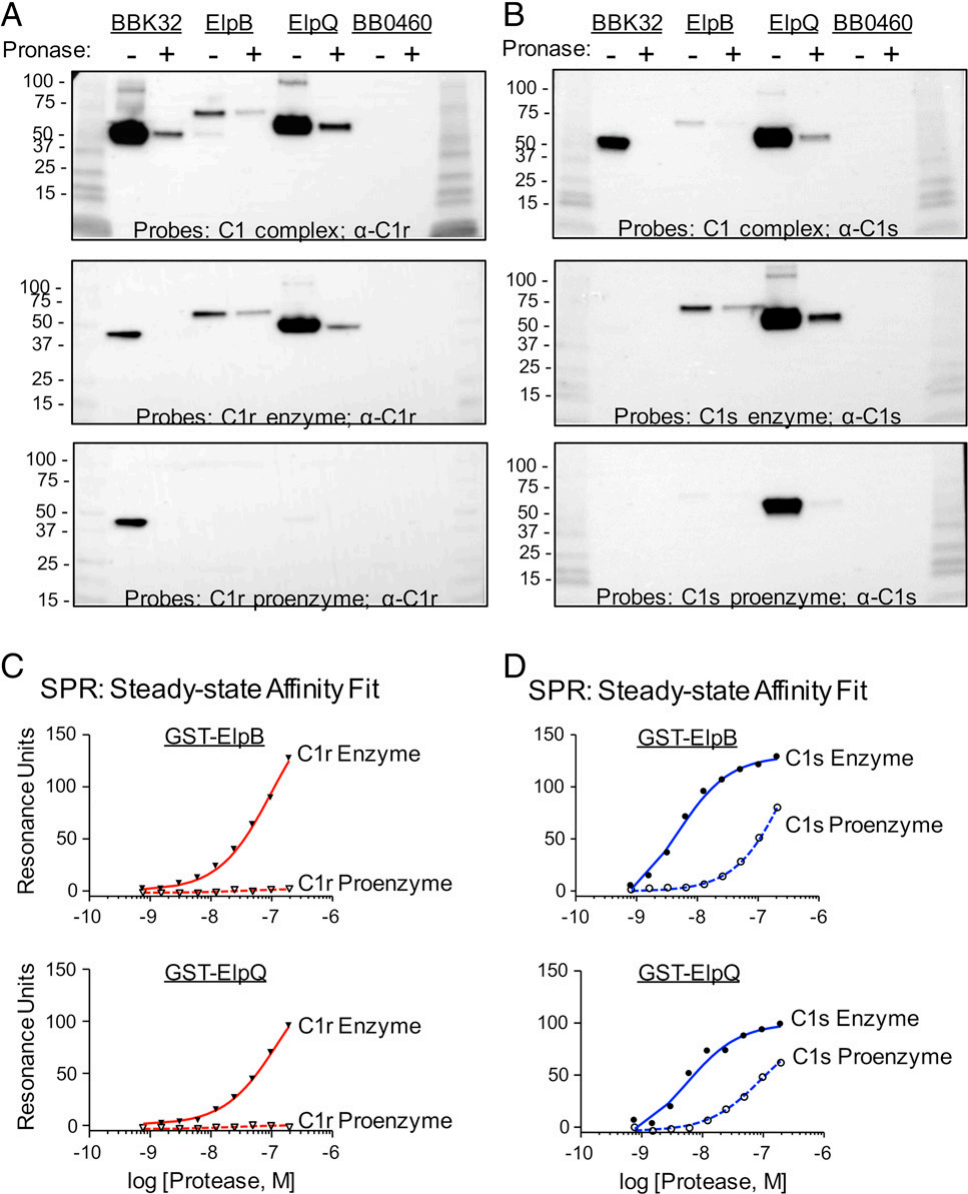
ElpB and ElpQ preferentially bind activated forms of C1r and C1s. (*A*) Extracts from untreated (“−”) or pronase-treated (“+”) 1 × 10^7^ strain B31-e2 spirochetes that ectopically produce the indicated surface lipoproteins were separated by SDS/PAGE and transferred to polyvinylidene difluoride (PVDF) membranes. The filters were probed with purified C1 complex (*Top*), activated C1r enzyme (*Middle*) or C1r proenzyme (*Bottom*), and bound probe revealed by anti-C1r antibody, followed by HRP-conjugated anti-mouse antibody. Shown is a representative of three experiments. (*B*) Filters prepared identically to *A* were probed with purified C1 complex (*Top*), activated C1s enzyme (*Middle*) or C1s proenzyme (*Bottom*), and bound probe revealed by anti-C1s antibody, followed by HRP-conjugated anti-mouse antibody. Shown is a representative of three experiments. (*C* and *D*) Biosensors immobilized with GST-ElpB (*Upper*) or GST-ElpQ (*Lower*) were tested by SPR for binding to the indicated concentrations of the enzyme or proenzyme forms of C1r (*C*) or C1s (*D*). Injection series were each performed in triplicate. For both *C* and *D*, steady-state affinity fits were determined by T200 Biacore Evaluation software and *K*_D_ values are reported in Table 1.

Finally, we probed these bacterial lysates using purified human C1 or the C1 subcomponent proteases to test for potential protein–protein interactions. Lysates from spirochetes expressing BBK32 (a C1r-binding positive control) contained a species that bound strongly to C1 complex, C1r proenzyme, and C1r enzyme but, as expected, to neither form of C1s (Fig. 2 *A* vs. *B*). In all cases the C1/C1r-binding species comigrated with epitope-tagged BBK32 (*SI Appendix*, Fig. S4*A*). The negative-control BB0460 lysates contained no species that bound detectably to any complement protein probe (Fig. 2 *A* and *B*). Consistent with the data shown in Fig. 1 and *SI Appendix*, Fig. S3, single bands coincident with ElpB and ElpQ, as judged by an α-6xHis blot (*SI Appendix*, Fig. S4*A*), bound to C1 complex, C1r enzyme (i.e., activated C1r), and C1s enzyme (i.e., activated C1s) (Fig. 2 *A* and *B*, *Top* and *Middle*). Furthermore, this binding was reduced in the lysates of cells treated with pronase.

Interestingly, we found that C1r proenzyme failed to bind either ElpB or ElpQ spirochete lysates (Fig. 2*A*, *Bottom*). C1s proenzyme also showed lower relative binding to ElpB compared to the activated form of C1s but due to the high levels of ElpQ production, comparison of relative binding of this protein to the proenzyme and activated forms of C1s was more difficult to assess (Fig. 2*B*, *Middle* and *Bottom*). To quantitatively investigate these interactions, we measured the relative affinities of pro- and active forms of both C1r and C1s for recombinant GST-ElpB and GST-ElpQ by SPR. Indeed, while GST-ElpB and GST-ElpQ bound to C1r enzyme with *K*_D_ values of 100 nM and 97 nM, respectively, neither protein exhibited detectable binding for C1r proenzyme (Fig. 2*C* and *SI Appendix*, Fig. S5). Similarly, GST-ElpB and GST-ElpQ bound C1s enzyme with ∼70-fold and ∼38-fold higher affinity, respectively, than C1s proenzyme (*K*_D_ = 3.9 nM vs. 270 nM; *K*_D_ = 4.5 nM vs. 170 nM) (Fig. 2*D*, Table 1, and *SI Appendix*, Fig. S5).

### ElpQ Inhibits C1s Cleavage of C2 and C4.

Having established that ElpB and ElpQ were capable of direct interaction with human C1 via specific recognition of the protease subcomponents, using ElpQ we explored a potential mechanism of action for C1 inhibition. To facilitate clarity in our gel-based cleavage assays and to eliminate the GST-tag from the mechanistic analysis, we generated an ElpQ construct lacking this epitope. The “tagless” ElpQ behaved nearly identically in SPR C1s-binding assays and ELISA-based complement assays when compared to GST-ElpQ (*SI Appendix*, Fig. S6).

**Fig. 3.**
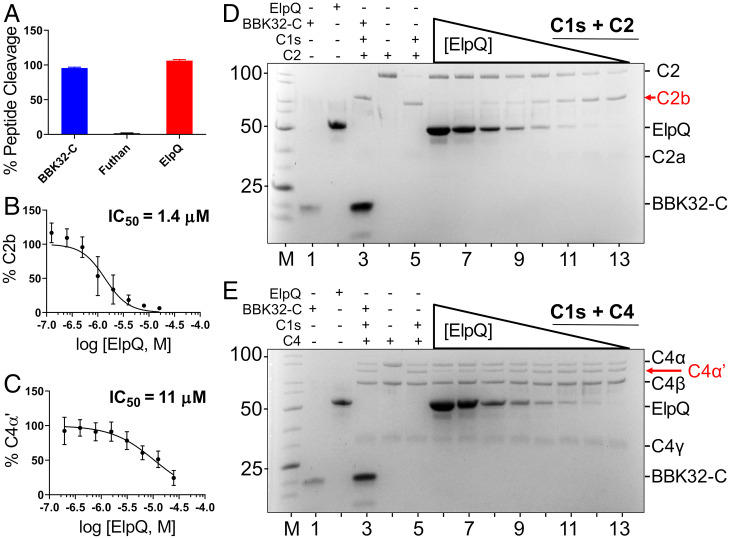
ElpQ inhibits the proteolytic activity of complement C1s. (*A*) Enzymatic cleavage by C1s of the small peptide substrate Z-L-Lys-sBzl was assayed with 5,5′-dithiobis-(2-nitrobenzoic acid) (Ellman’s reagent) in the presence of 25 μM BBK32-C (noninhibitory control) or ElpQ at 25 °C for 1 h. Experiments were performed in triplicate. Absorbance was read at 412 nm and signals were normalized to negative control no-substrate wells. (*B* and *D*) Proteolytic cleavage of C2 by C1s enzyme produces ∼70 kDa C2b and ∼35 kDa C2a after 1 h at 37 °C. Lanes 1 to 5: Control reactions in the presence (“+”) or absence (“−”) or 25 µM ElpQ, 25 µM BBK32-C (noninhibitory control), 6.25 nM C1s, and 685 nM C2. (Note that the amount of C1s loaded is below the level of detection by SDS/PAGE). (*D*, lanes 6 to 13) C2b accumulation in the presence of 6.25 nM C1s, 685 nM C2 and a twofold dilution series (from 16 to 0.13 μM) of ElpQ. In *B*, the fraction of C2b relative to total input C2 in the same lane determined by densitometry analysis data are normalized to C2 (lane 4) and C1s digested C2 (lane 5). A representative gel is shown. The experiment was performed three times. (*C* and *E*) C4, which consists of three polypeptide chains—C4α (97 kDa), C4β (77 kDa), C4γ (33 kDa)—is cleaved by C1s enzyme for 1 h at 37 °C to produce C4α′ (88 kDa). (*E*, lanes 1 to 5) SDS/PAGE profile in the presence (“+”) or absence (“−”) or 25 µM ElpQ, 25 µM BBK32-C (noninhibitory control), 6.25 nM C1s, and 616 nM C4. (Lanes 6 to 13) SDS/PAGE profile in the presence of 6.25 nM C1s, 616 nM C4, and a twofold dilution series (from 25 to 0.20 μM) of ElpQ. In *C*, the fraction of C4α′ relative to input C4β in the same lane and normalized to C1s + C4 positive-control (lane 5) and negative-control C4 (lane 4) was determined by densitometry analysis.

BBK32, which binds to C1r but not C1s, is capable of directly inhibiting purified C1r enzyme cleavage of C1s proenzyme (34). In contrast, recombinant ElpQ failed to block this reaction at protein concentrations several orders of magnitude greater than the C1r/ElpQ *K*_D_ (*SI Appendix*, Fig. S7*A*). ElpQ also failed to prevent the cleavage of the small peptidic C1r substrate Z-Gly-Arg-sBzl (52), whereas BBK32 did so readily (*SI Appendix*, Fig. S7*B*). Similarly, unlike futhan, a small molecule active site C1s inhibitor (52), 25 µM ElpQ (i.e., >5,500-fold over the measured *K*_D_) (Table 1) failed to inhibit the cleavage of the C1s peptidic substrate Z-L-Lys thiobenzyl by C1s (Fig. 3*A*). Thus, in the C1s/ElpQ complex, the active site of C1s remains accessible to a small peptide substrate.

We next tested whether ElpQ was capable of inhibiting C1s-mediated cleavage of native substrates. The cleavage of C2 or C4 by purified C1s was monitored by SDS/PAGE in the presence of increasing concentrations of ElpQ (Fig. 3 *B–E*). Whereas BBK32 failed to block C2 cleavage by C1s (Fig. 3*D*, lane 3), as judged by the generation of the C2 cleavage product C2b (“← C2b”) (Fig. 3*B*), ElpQ blocked C1s-mediated C2 proteolysis and the concomitant formation of C2b in a dose-dependent fashion (Fig. 3*D*, lanes 6 to 13). Likewise, while BBK32 failed to prevent C4 cleavage by C1s (Fig. 3*E*, lane 3), as judged by generation of the C4 cleavage product C4α′ (“← C4α′”) (Fig. 3*C*), ElpQ did so in a dose-dependent manner (Fig. 3*E*, lanes 6 to 13). Densitometry analysis resulted in calculated ElpQ IC_50_ values of 1.4 µM and 11 µM for C2 and C4, respectively. Similarly, a tagless version of ElpB blocked C2 cleavage by C1s, and with an IC_50_ (0.87 µM) like that of ElpQ (*SI Appendix*, Fig. S8). The observation that ElpQ inhibited the cleavage of large endogenous C1s substrates but not a small peptide C1s substrate suggests that ElpQ inhibits C1s in a manner that leaves the active site of C1s accessible to small peptides.

### ElpQ Inhibits the CP of Complement.

**Fig. 4.**
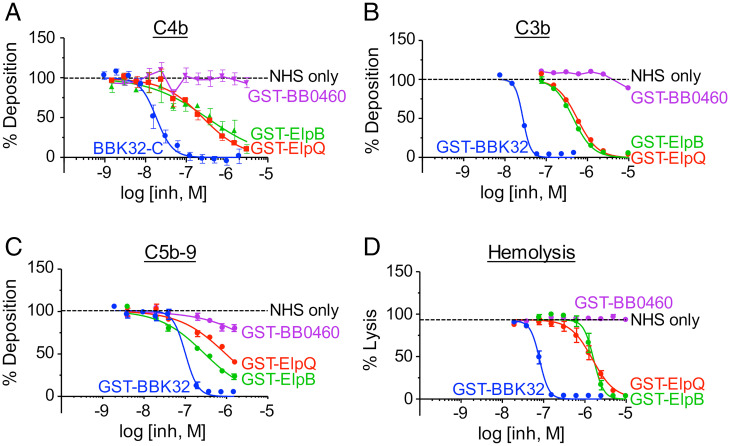
ElpB and ElpQ inhibit the CP of complement. (*A–C*) Normal human serum (NHS) was incubated with the indicated concentration of purified GST-fusion proteins, then added to wells precoated with human IgM. Deposition of (*A*) C4b, (*B*) C3b, or (*C*) C5b-C9 was determined by the addition of the appropriate primary and secondary antibodies (*Materials and Methods*) enumerated by absorbance at OD_405nm_ or OD_450nm_. Each well was normalized to wells with no inhibitor (100%) and no serum (0%). Curves were fit using nonlinear regression to determine IC_50_ values. (*D*) NHS was incubated with the indicated concentration of purified GST-fusion proteins and then added to preopsonized sheep erythrocytes (*Materials and Methods*). Erythrocyte lysis was determined by OD_405nm_ and normalized to lysis by deionized water (100%) and no serum (0%). Error bars indicate SEM. Each concentration was tested a minimum of three times.

Collectively, the data above identify an interaction between surface-expressed *B. burgdorferi* lipoproteins ElpB and ElpQ with human C1 and demonstrate that recombinant ElpQ blocks C1s activity. The CP is initiated by this C1 activity, so we tested the ability of ElpB and ElpQ to block successive steps in this pathway. Recombinant GST-ElpB or GST-ElpQ fusion proteins were added at increasing concentrations to normal human serum in microtiter wells coated with IgM to initiate CP activation. The surface deposition of C4b, C3b, and C5b-9, mimicking the fixation of successive components of the CP (31), was measured by ELISA. GST-BBK32 and GST-BB0460 served as positive and negative controls, respectively. Both GST-ElpB and GST-ElpQ inhibited the deposition of these three components in a dose-dependent manner, with half-maximal inhibitory concentrations (IC_50_s) approximately 10-fold higher than the IC_50_ of GST-BBK32 (Fig. 4 *A*–*C* and Table 2). GST-BB0460 showed no inhibitory activity. As C5b-9 is the MAC, capable of generating pores in membranes, we further tested each protein for protection of antibody-sensitized sheep red blood cells from CP-mediated lysis. As above, GST-ElpQ and GST-ElpB inhibited lysis in a dose-dependent manner, with an IC_50_ of 1.5 and 1.6 μM, respectively, or ∼20-fold higher than the IC_50_ of GST-BBK32 (Fig. 4*D* and Table 2).

**Table 2.** IC_50_ for ElpB- and ElpQ-mediated inhibition of the CP of complement

Protein	Hemolysis (nM)	C5b-9 (nM)	C3b (nM)	C4b (nM)*
GST-BBK32	79 ± 5.2	100 ± 20	29 ± 1.0	18 ± 3.3
GST-ElpQ	1500 ± 240	1000 ± 210	450 ± 26	260 ± 62
GST-ElpB	1600 ± 190	300 ± 43	570 ± 29	330 ± 130

* IC_50_ based on non-GST-tagged BBK32-C construct.

### Ectopic Production of ElpQ Protects Spirochetes from Complement-Mediated Killing.

The ability of recombinant GST-ElpB and GST-ElpQ to block complement deposition products and prevent lysis of red blood cells by the MAC suggested that these proteins may protect spirochetes from antibody-dependent complement attack. We tested the ability of *B. burgdorferi* B31-e2 strains that ectopically produce (His-tagged) ElpB or ElpQ to resist CP killing, with BBK32 and BB0460 as positive and negative controls, respectively. Based on a previously described assay to initiate the CP (53), we incubated these strains with either anti-*B. burgdorferi* polyclonal antibodies or nonspecific antibodies (as a negative control), then added normal human serum to provide complement components and lysozyme to facilitate disruption of spirochetal integrity (*Materials and Methods*). After dilution into BSK-II media and 72-h incubation to allow for growth of surviving bacteria, we enumerated living spirochetes. Although the ElpQ-producing strain did not grow quite as well after exposure to nonspecific antibodies and lysozyme, all the four strains grew to high titers (i.e., 1.6 × 10^7^/mL and 1.1 × 10^8^/mL), indicating that the lysozyme treatment alone was insufficient to kill bacteria efficiently (*SI Appendix*, Table S3). We speculate that the high-level ectopic overproduction of protein by the ElpQ producer may slightly diminish the overall fitness of this strain.

**Fig. 5.**
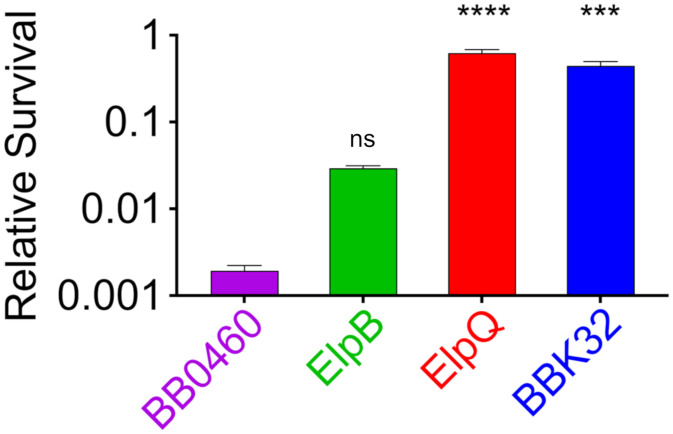
Ectopic production of ElpQ protects spirochetes from complement-mediated killing. 5 × 10^7^ spirochetes were treated with a 4 μg/mL of an α-*B. burgdorferi* strain B31 mouse polyclonal antibody or its isotype (IgG) control, followed by exposure to 20% untreated or heat-inactivated NHS containing 10 μg/mL lysozyme for 4 h. Samples were grown in BSK-II for 72 h at 33 °C and enumerated by dark-field microscopy. Relative survival was calculated by dividing the average culture density of triplicate samples of the α-*B. burgdorferi* experimental group by the average density of the control Ab group for each strain. Shown is the mean and SEM of triplicate samples from a representative of two experiments. Comparisons of significance were made between BB0460 control and the three test strains. ****P < 0.001; ***P < 0.001; ns, not significant using one-way ANOVA with Dunnett’s multiple comparisons test.

To assess the ability of ElpB and ElpQ to counter classical complement killing, we calculated relative survival: that is, the titer of spirochetes treated with anti-*B. burgdorferi* polyclonal antibodies normalized to the titer of spirochetes treated with nonspecific antibodies. As predicted, a *B. burgdorferi* B31-e2 high-passage strain that ectopically produced BB0460 was highly susceptible to antibody-dependent complement killing, with a relative survival of less than 0.002 (Fig. 5, purple). This degree of killing required not only anti-*B. burgdorferi* antibody, but was abrogated by prior heat treatment of serum to inactivate complement (*SI Appendix*, Table S3). Conversely, production of BBK32 conferred high-level protection, with relative survival of 0.45, or ∼230-fold higher than the negative control BB0460 (*P* < 0.001) (Fig. 5, blue). *B. burgdorferi* B31-e2 producing ElpQ displayed a relative survival of 0.62, 321-fold higher than the control (*P* < 0.0001) (Fig. 5, red). *B. burgdorferi* B31-e2 expressing ElpB, which appeared to produce a small fraction of ectopic protein compared to the ElpQ-producing strain when assessed by immunoblotting (Fig. 1 *A* and *B*), exhibited a relative survival of 0.03, 15-fold higher than control, but this difference did not reach statistical significance (Fig. 5, green). Nevertheless, the dramatically enhanced resistance to CP-mediated killing conferred by ElpQ, along with the trend in protection by the ElpB-producing strain, indicates that the inhibition of C1 and blockade of the CP observed in biochemical assays reflects an activity that protects bacterial viability.

## Discussion

Lyme disease spirochetes are typical of other spirochetal pathogens in that they encode many lipoproteins (11). Although the proportion of lipoproteins located in the periplasm varies among spirochetes (8, 10, 54, 55), surface lipoproteins are critical to pathogenesis and provide an important means by which pathogenic spirochetes interact with the host environment (56, 57). Of the ∼125 lipoproteins encoded by the *B. burgdorferi* genome, the majority localize to the outer membrane (10), although functions for relatively few of these proteins have been elucidated. Adding to the complexity of understanding lipoprotein function, several of the best-characterized *B. burgdorferi* outer surface lipoproteins, such as OspC and BBK32, have been shown to provide multiple independent functions during murine borreliosis (58–62). Building on the generation of a comprehensive lipoprotein library (10), we developed an ectopic overexpression screening methodology to identify novel interactions between host macromolecules and the *B. burgdorferi* surface lipoproteome expressed in its native environment in the outer membrane of intact spirochetes. This methodology has the potential to uncover diverse host interactions that take place at the spirochete surface and may be valuable in the study of other pathogenic bacteria as well.

A clear limitation of the “gain-of-function” strategy described here is that overproduction of ectopically expressed lipoproteins may uncover artifactual functions. Indeed, to investigate functional aspects of the lipoproteome most easily, the *B. burgdorferi* B31-e2 gain-of-function library was constructed using the flagellin promoter, P_flaB_, one of the strongest constitutive *B. burgdorferi* promoters (63). Although we did not quantify levels of ectopic lipoprotein production in this study, immunoblotting revealed that ElpQ was produced in vastly greater amounts than ElpB, with the likely consequence that only the ElpQ producer was shown to protect strain B31-e2 significantly from classical complement killing (Fig. 5). This limitation notwithstanding, ectopic overexpression for gain-of-function analyses have identified and analyzed the function of many *B. burgdorferi* adhesive lipoproteins that have been subsequently validated by genetic studies involving infectious *B. burgdorferi* strains (61, 64–68).

We applied the current lipoproteome screening strategy to the requirement that, as an extracellular pathogen that encounters host blood during both the tick bloodmeal and throughout dissemination and colonization of their vertebrate hosts, Lyme disease spirochetes must prevent complement-mediated opsonization and lysis at multiple stages in the enzootic cycle. Moreover, the complement system employs three distinct pathways for activation that together form a complex host defense. Reflecting this, nearly a dozen different *B. burgdorferi* outer surface lipoproteins have been shown to directly interact with complement components, disrupting their activities (13, 14). At least three factors contribute to the multiplicity of lipoproteins devoted to thwart complement defense. First, distinct borrelial complement evasion proteins block different complement activation pathways. For example, BBK32 selectively targets C1r, the initiator protease of the CP, while OspC binds to C4b, the downstream activation product of both the classical and lectin pathways (20, 34, 35). Second, individual borrelial lipoproteins may target the same host protein but function at different stages of the enzootic cycle. *B. burgdorferi* CspA and CspZ both bind to factor H and prevent activation of the alternative pathway, but CspA is expressed exclusively in the tick midgut and prevents the bactericidal effects of the bloodmeal, whereas CspZ is produced early in vertebrate infection and fosters the establishment of infection in that host (32, 33, 69). Finally, although some Lyme disease spirochete strains are restricted to only a single vertebrate, other strains have the capacity to infect multiple vertebrate hosts (70) that encode polymorphic complement components (71, 72). Indeed, variation in CspA sequences has been shown to dictate binding to mammalian vs. avian factor H and the concomitant capacity to infect these two hosts (33, 73). Likewise, the production of multiple complement-inactivating proteins may permit the broad host specificity displayed by some *B. burgdorferi* strains. Thus, the collective activities of multiple complement evasion proteins of *B. burgdorferi* may provide the distinct temporal and spatial needs to thrive in enzootic cycles that involve multiple hosts. Due to the complexity of these interactions. *B. burgdorferi* serves as a useful model for understanding how a wide range of complement inactivation mechanisms together foster the retention of a pathogen in nature.

Consistent with the observation that partial functional redundancy is a hallmark of the *B. burgdorferi* complement evasion system, BBK32 was sufficient to protect spirochetes from complement-mediated killing, but *bbk32*-deficient mutants remained serum-resistant (34). Thus, we focused our surface lipoproteome screen on the CP component C1. We identified two members of the paralogous Elp protein family, ElpB and ElpQ (from *B. burgdorferi* strain B31), as capable of forming high-affinity interactions with the human C1 complex (Figs. 1 and 2 and *SI Appendix*, Fig. S3). The Erp family encompasses more than 17 genes in strain B31 (51) that share highly homologous leader peptides and DNA sequence at the 5′ end of their operons (23, 41, 42). However, as discussed above, the amino acid sequences of their mature proteins group them into the evolutionarily unrelated OspE-related proteins, many of which have been shown to bind factor H (74–78), OspF-related proteins, which bind to heparan sulfate (32), and the Elp subfamilies (41). Our finding that two Elp members bind to complement C1 further emphasizes the divergent functions among the three subfamilies (41).

**Fig. 6.**
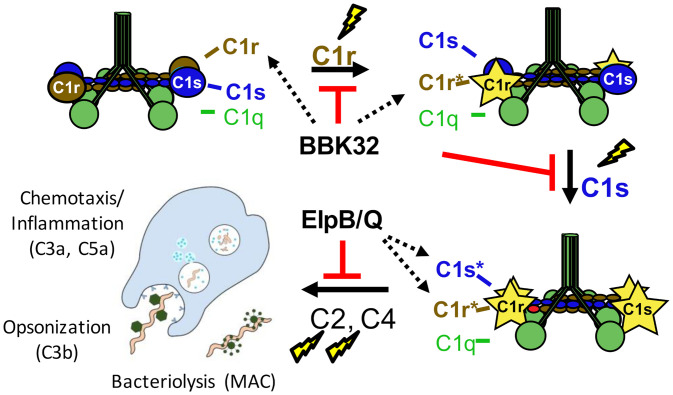
BBK32 and ElpB/Q inhibit C1 by distinct mechanisms. Following binding of complement C1 to the spirochete surface via C1q, C1 activation proceeds through three cleavage steps (lightning bolts): 1) C1r (brown) is autoactivated from its zymogen to active form (C1r*; yellow star), followed by 2) C1r cleavage of C1s (blue) from its zymogen to active form (C1s*; yellow star), leading to 3) C1s cleavage of C2 and C4, and complement activation with associated release of anaphylatoxins (i.e., C3a, C5a), opsonization of spirochetes leading to phagocytosis (i.e., C3b), and bacteriolysis (i.e., MAC). BBK32 binds to both the zymogen C1r and active C1r forms (dotted arrows), resulting in direct blockade of either the autocatalytic event or C1r-mediated C1s cleavage. In contrast, ElpB and ElpQ bind specifically to the activated forms of both C1r and C1s (dotted arrows), preventing cleavage of C2 and C4 by activated C1s protease.

Consistent with the mechanistic divergence of anticomplement lipoproteins, ElpB and ElpQ, like BBK32, prevent antibody-mediated complement activation but target the C1 complex via distinct means. BBK32 does not bind C1s, but recognizes both zymogen and activated forms of C1r, blocking its enzymatic activity. In contrast, ElpB and ElpQ bind to both C1r and C1s but selectively recognize activated forms of the proteases (Fig. 2 and *SI Appendix*, Fig. S5). Furthermore, we showed that ElpQ is incapable of directly blocking purified C1r activity (*SI Appendix*, Fig. S7) but prevents cleavage of both C2 and C4 by purified activated C1s enzyme (Fig. 3 *B* and *C*); ElpB possesses a similar activity (*SI Appendix*, Fig. S8). Finally, ElpQ did not prevent cleavage of small peptide substrates, and is unusual among microbial-derived serine protease inhibitors, many of which—such as ecotin or BBK32 (79)—target the protease active site (80, 81). A model detailing the mechanistic differences between BBK32 and ElpB/Q C1 inhibition is presented in Fig. 6.

Previous work showed that expression of BBK32 by a high-passage, noninfectious *B. burgdorferi* strain enhanced serum resistance, and that simultaneous inactivation of the classical and lectin pathways eliminated this enhancement, indicating that BBK32 blocked one or both pathways. To confirm that the C1-binding activities of BBK32, ElpB, and ElpQ specifically blocked classical complement killing, we triggered this pathway by treating high-passage strains that ectopically produce these proteins with anti*-B. burgdorferi* antibody. Whereas BBK32, ElpB, and ElpQ provided no survival advantage when spirochetes were treated with serum supplemented with isotype control antibody, all three lipoproteins promoted survival when incubated with specific antibody, indicating that the C1r- or C1s-inhibitory activities of BBK32 or ElpB and ElpQ, respectively, protected spirochetes from classical complement killing. ElpQ provided the greatest degree of protection, enhancing the survival ratio 321-fold relative to BB0460, compared to 230-fold and 15-fold for BBK32 and ElpB, respectively (Fig. 5). Notably, BBK32 and ElpQ appeared to be expressed at much higher levels than ElpB (Fig. 2*A*). In addition, BBK32 inhibited C4b and C3b in vitro deposition and complement-mediated red blood cell hemolysis at ∼10-fold lower concentrations than ElpB or ElpQ (Fig. 5).

The innate and adaptive immune system intersect at the level of the CP of complement when antibody–antigen immune complexes are recognized by complement C1, triggering the complement cascade. Both ElpB and ElpQ are antigenic during experimental murine and human infection, indicating that they are produced in vivo and consistent with the hypothesis they function during mammalian infection (23, 82, 83). Blocking complement C1 may be critical for *B. burgdorferi* persistence in immunocompetent hosts, which generate a specific antibody response during chronic infection. This activity might also be required to establish infection in a previously infected host or, given that natural antibodies recognize the Lyme disease spirochete (84), in a naïve host.

ElpB and ElpQ display highly similar biochemical activities, and no evidence to date has indicated divergent expression patterns between the two genes, raising the possibility that they are functionally redundant. In addition, other Elp family members, such as ElpX and ElpM (which is as closely related to ElpB and ElpQ as they are to each other) (*SI Appendix*, Table S4), did not bind human C1 in our lipoproteome library screen (Fig. 1*A*). Complement C1 is polymorphic among vertebrates, and whether these Elps recognize C1 of other *B. burgdorferi* hosts, perhaps contributing to host specificity, remains to be tested (85). Given the potential functional redundancy of subsets of Elp proteins, definitive experimental assessment of their role during infection is likely to require mutation or silencing of multiple *elp* genes in an otherwise infectious strain background. For example, strain B31-A3, a strain that is commonly utilized for genetic studies, encodes, in addition to ElpQ, three proteins identical to ElpB (23, 50). Moreover, Elp proteins likely function in the context of other anticomplement factors—such as BBK32, OspC, or OspE family members—that together inactivate multiple complement pathways to facilitate survival in the host. This complexity will require genetic techniques not routinely performed for *B. burgdorferi* at this time, but recent description of a CRISPR interference platform for silencing *B. burgdorferi* genes may facilitate these important investigations (86).

## Materials and Methods

### Bacterial Strains, Plasmids, and Lipoprotein Gain-of Function Library.

*Escherichia coli* strains DH5α and BL21(DE3) were used for plasmid cloning and propagation and protein purification, respectively, as cultured in LB-Miller broth, as described in *SI Appendix*, *Supplemental Materials and Methods*. An epitope-tagged *B. burgdorferi* lipoprotein expression (gain-of-function) library in the high-passage, noninfectious B31-e2 background strain (10) (*SI Appendix*, Table S1) was grown in supplemented BSK-II medium, as described in *SI Appendix*, *Supplemental Materials and Methods*. Plasmids are described in *SI Appendix*, Table S1 and primers to generate several plasmids are described in *SI Appendix*, Table S5.

### Quantitation of Binding of Gain-of-Function Library Clones to Immobilized Substrates.

Binding of gain-of-function library clones to immobilized substrates was measured using a modification of a previously described ELISA-based assay (87), as described in *SI Appendix*, *Supplemental Materials and Methods*.

### Quantitative ELISA or SPR to Assess *B. burgdorferi* Lipoprotein Binding to Purified Human C1 Components.

To quantitate the ability of *B. burgdorferi* lipoproteins to bind purified components of the C1 complex, we adapted a previously described quantitative ELISA-based (65) or SPR-based assays, using a Biacore T200 (GE Healthcare), as previously described (34), with modifications as described in *SI Appendix*, *Supplemental Materials and Methods*.

### Inhibition of C3d, C4d, C5b-9 Deposition, and Erythrocyte Hemolysis by Recombinant *B. burgdorferi* Lipoproteins.

We adapted previously described ELISA-based assays to determine the effect of recombinant *B. burgdorferi* lipoproteins on CP-mediated deposition of C3d (34, 88) or C4d (34). Inhibition of CP-mediated erythrocyte hemolysis by recombinant *B. burgdorferi* lipoproteins was assayed using a modified version of the previously described CP hemolytic assay (12, 34, 89), as described in *SI Appendix, Supplemental Materials and Methods*.

### Inhibition of C1r and C1s Enzyme Activity by Synthetic Peptide Cleavage.

C1r enzyme assays were achieved by monitoring the autolytic activation of C1r proenzyme using the substrate Z-Gly-Arg-sBzl, and C1s enzyme assays using Z-L-Lys-sBzl, as described in *SI Appendix*, *Supplemental Materials and Methods*.

### Gel-Based Assays to Detect Inhibition of C1r-Mediated Proenzyme C1s Cleavage or C1s-Mediated C2/C4 Cleavage.

Enzymatic inhibition assays were performed using SDS/PAGE to monitor the cleavage of proenzyme C1s, as previously described, with modifications described in *SI Appendix*, *Supplemental Materials and Methods* (34). Similarly, to assess inhibition of C1s mediated cleavage of C2 or C4, C2- or C4-derived cleavage products were detected by SDS/PAGE after incubation of substrates with twofold dilutions of ElpQ or ElpB, as described in *SI Appendix*, *Supplemental Materials and Methods*.

### CP-Mediated Serum-Killing Assay

The ability of *B. burgdorferi* B31-e2 strains to resist CP killing was based on a previously described assay that involved anti-*B. burgdorferi* polyclonal (or control) antibodies, human serum to provide complement, and lysozyme to facilitate disruption of spirochetal integrity of MAC-associated bacteria (53). After treatment, cultures were grown for 72 h and spirochetal titers were counted in duplicate by dark-field microscopy (*SI Appendix*, *Supplemental Materials and Methods*).

## Supplementary Material

Supplementary File

## Data Availability

All study data are included in the article and *SI Appendix*.
