# Supplementary material for: Lipoproteome screening of the Lyme disease agent identifies inhibitors of antibody-mediated complement killing
Source: Proc Natl Acad Sci U S A. 2022 Mar 21;119(13):e2117770119. doi: 10.1073/pnas.2117770119 (PMC9060444; doi:10.1073/pnas.2117770119)
Supplement: Supplementary File [file pnas.2117770119.sapp.pdf]

Supplemental Information for "Lipoproteome screening of the Lyme disease agent identifies novel inhibitors of antibody-mediated complement killing"

Michael J. Pereira[a], Beau Wager[a], Ryan J. Garrigues[b], Eva Gerlach[c], Joshua D. Quinn[a], Alexander S. Dowdell[d], Marcia S. Osburne[a], Wolfram R. Zückert[d], Peter Kraiczy[c], Brandon L. Garcia[b,*] John M. Leong[a,*]

[a] Department of Molecular Biology and Microbiology, Tufts School of Medicine, Tufts University, Boston, Massachusetts, USA

[b] Department of Microbiology and Immunology, Brody School of Medicine, East Carolina University, Greenville, North Carolina, USA

[c] Institute of Medical Microbiology and Infection Control, University Hospital of Frankfurt, Goethe University Frankfurt, D-60596 Frankfurt, Germany.

[d] Department of Microbiology, Molecular Genetics, and Immunology, University of Kansas Medical Center, Kansas City, KS, USA

MP and BW contributed equally to this work

*Corresponding author: JML, BLG

Email: John.Leong@tufts.edu; garciabr18@ecu.edu

This PDF file includes:

Supplementary text

Figures S1 to S8

Tables S1 to S5

SI References

Supplemental Materials and Methods

Description of lipoprotein gain-of-function library and culture of B. burgdorferi.

The gain-of-function library consists of 80 individual clones in the high-passage, non-infectious strain *B. burgdorferi* B31-e2 containing the low copy (approximately 10 copies per cell) pSC:LP vector, where LP represents a unique epitope-tagged, surface-exposed *B. burgdorferi* lipoprotein expressed in each of the 80 clones (1); **Table S1**). Ectopic expression of each lipoprotein-encoding gene is driven by the *B. burgdorferi flaB* constitutive promoter. *B. burgdorferi* strains were grown in BSK-II medium supplemented with 6% (v/v) heat-inactivated normal rabbit serum (Atlanta Biologicals) at 33°C, pH 7.6, with ambient levels of CO_2 (2). BSK-II was supplemented with 100 µg/ml of kanamycin (Sigma Aldrich) as necessary. The library was arrayed in multiple 96-well, sterile, flat bottom plates at a concentration of 1×10^7 spirochetes/well (1×10^8 per ml) and stored at -80°C in BSK-II with 20% (v/v) glycerol.

Quantitation of binding of gain-of-function library clones to immobilized substrates

One µg/well purified BSA (Sigma Aldrich), or human derived fibronectin (Corning) or C1 complex (Complement Technologies) in coating buffer [0.1M sodium bicarbonate, pH 9.6] was used to coat wells of a 96-well ELISA plate (Nunc Maxisorp) at 4°C overnight. On the same day, a single 96-well plate containing the gain-of-function library was thawed at room temperature (RT) and centrifuged (1,250 × g, 15 min, RT). The supernatant was discarded, and the spirochetes were resuspended in 200 µl/well of BSK-II and allowed to recover under normal growth conditions. The following day, 80 µl (~4×10^6 cells) of culture from each well of the 96-well plate were transferred to a new plate and centrifuged (1,250 × g, 15 min, RT). The supernatant was discarded, and the cells were resuspended in 200 µl/well HBS-DB [25 mM HEPES, 105 mM sodium chloride, 1 mM manganese chloride, 1 mM magnesium chloride, 0.1% (w/v) dextrose, 0.2% (w/v) BSA, pH 7.8)].

The previously coated 96-well plate was washed three times with PBS-T [10 mM disodium phosphate, 1.8 mM monopotassium phosphate, 137 mM sodium chloride, 2.7 mM potassium chloride, 0.05% (v/v) Tween-20, pH 7.4] and blocked with 200 µl/well of Ultrablock (BioRad) for 1.5 hours.

The blocking buffer was discarded and the plate was washed, then inoculated with 50 µl/well (~1×10^6 spirochetes) of the resuspended gain-of-function library, then centrifuged (1,250 × g, 15 min, RT) to facilitate spirochete-substrate interaction. After one hour at RT and three washes to remove unbound spirochetes, bound spirochetes were fixed with 4% formaldehyde (v/v) for 20 minutes at RT. The formaldehyde was removed and plates were air dried on the bench top overnight. Fixed spirochetes were permeabilized with 50 µl/well of ice-cold methanol for 10 minutes at -20°C. Methanol was removed and the plates were air dried for several minutes. Wells were blocked with 200 µl/well of 5% (w/v) non-fat dry milk in PBS-T for one hour, followed by washing.

To detect spirochete binding, wells were incubated with a 1:800 dilution of a polyclonal rabbit α-Bb antibody (Abcam, ab20118) for one hour at RT, then washed and probed with a 1:2,000 dilution of an α-rabbit alkaline phosphatase conjugated antibody (Sigma Aldrich, cat # A3687) for one hour at RT. Wells were washed and signal developed using the SigmaFast pNpp reagent (Sigma Aldrich). OD_{405nm} was determined every minute for 15 minutes using a BioTek Synergy HT plate reader and Gen5 software. Bacterial binding is expressed as the Vmean of ΔOD_{405nm}, calculated by determining the slope of the [OD_{405nm} vs. time] best fit line across the linear portion of the 15-minute kinetic assay. All experiments were repeated at least twice.

Pronase treatment, conventional western and far western immunoblotting

Surface proteolysis of expressed lipoproteins was performed as previously described (3). Briefly, 1×10^8 spirochetes were washed three times in HBS-DB and resuspended in 100 µl of HBS-DB. Spirochetes were then treated with 40 µg/ml (final concentration) of pronase (Sigma) for one hour at room temperature. Reactions were inactivated with 2 mM phenylmethanesulfonyl fluoride (PMSF) (Sigma Aldrich) and cells were lysed by boiling for ten minutes in Laemmli buffer (3). Bacterial lysates were resolved by SDS-PAGE on a 4-20% gradient polyacrylamide gel at 75 V for 1.5 hours. After electrophoresis, samples were transferred to a PVDF membrane and used for western immunoblotting.

Conventional western immunoblotting was performed by blocking the PVDF membrane with 5% (w/v) nonfat dry milk in PBS-T. Antibodies used include the CD-1 antibody (a generous gift from Jorge Benach, Stony Brook University) (1:1000 dilution) for the detection of *B. burgdorferi flaB*, and a combination of α-6×His antibody (Sigma Aldrich, H1029) and HisProbe-HRP (Thermo) for the detection of affinity-tagged lipoproteins. Following washing, immunoblots were probed with the appropriate secondary α-mouse antibody conjugated to horseradish peroxidase (Promega, W402B) at a dilution of 1:5000. Immune complexes were detected using the SuperSignal West Pico Chemiluminescent Substrate (ThermoFisher Scientific), following the manufacturer's instructions, and imaged using a Syngene G:Box XR5 imager.

Far western immunoblotting was performed as previously described (4). Briefly, membranes were blocked in 5% (w/v) nonfat dry milk in PBS-T, followed by incubation with 2 µg/ml of purified C1, C1r, or C1s (Complement Technologies) in protein binding buffer [20 mM Tris (pH 7.5), 0.1M sodium chloride, 1 mM EDTA, 1 mM DTT, 10% (v/v) glycerol, 0.1% (v/v) Tween-20, 5% (w/v) nonfat dry milk] overnight at 4°C. The next day, membranes were washed in PBS-T and were incubated with α-C1r (R&D Systems, MAB1807) or α-C1s (R&D Systems,

MAB2060) antibodies, as indicated, following the manufacturer's recommended dilutions for immunoblotting. Following washing, immunoblots were probed with the secondary α-mouse (C1r/s) (Promega, W402B) antibodies conjugated to horseradish peroxidase, at a dilution of 1:5000. Immune complexes were detected using the SuperSignal West Pico Chemiluminescent Substrate (ThermoFisher Scientific), according to the manufacturer's instructions, and imaged using a Syngene G:Box XR5 imager.

Expression plasmid cloning and protein purification

All primers used in this study are listed in **Table S5**. To generate expression plasmids encoding N-terminal GST fusions of ElpB, ElpQ, BBK32, and BB0460, genomic DNA was first prepared from these *B. burgdorferi* B31-e2 expression strains using the DNeasy Blood and Tissue Kit (Qiagen). The open reading frame (lacking the putative lipoprotein signal sequence) of each gene was PCR amplified using Q5 Hot Start Master Mix (New England Biolabs). Each PCR fragment, except for the one encoding ElpB, was engineered into the MCS of the pGEX4T2 expression vector (GE Healthcare Life Sciences) using BamHI and XmaI restriction sites. For *elpB*, which contains an internal BamHI restriction site, EcoRI and XmaI were used. Inserts were ligated into vector pGEX4T2 and the ligations were transformed into *E. coli* DH5α as previously described (5). Transformants were confirmed by BamHI/EcoRI and XmaI restriction digest of the plasmids, followed by gel electrophoresis on a 1% agarose gel for one hour at 75 V. Clones containing the correct insert were Sanger sequenced using an ABI 3130XL automated sequencer (Applied Biosciences). Confirmed plasmids were subsequently transformed into *E. coli* BL21(DE3) as previously described (5).

To purify GST-tagged proteins, *E. coli* BL21(DE3) cells encoding the appropriate plasmid were grown in LB-Miller broth (BD Difco), supplemented with 50 µg/ml of carbenicillin (Sigma

Aldrich) or 100 µg/ml of streptomycin (Sigma Aldrich) as appropriate, with aeration at 37°C to an OD600nm of 0.6, then induced with 1 mM IPTG (Sigma Aldrich) with aeration at room temperature overnight. The following day, cells were lysed using an M-110S Microfluidizer (Microfluidics) and proteins were purified using glutathione chromatography according to the manufacturer's instructions (GE Healthcare Life Sciences). To confirm the size and purity of purified recombinant protein, 25 µl of column eluate was resolved by SDS-PAGE on a 4-20% gradient polyacrylamide gel run at 75 V for 1.5 hours. Gels were then stained for 30 minutes with Coomassie blue solution [0.25% (w/v) Coomassie brilliant blue R-250, 45% (v/v) methanol, 10% (v/v) glacial acetic acid], rinsed in deionized water, and destained for two hours with destain solution [40% (v/v) methanol, 10% (v/v) glacial acetic acid]. Stained gels were imaged using a Syngene G:Box XR5 imager.

Untagged $ElpQ_{19-343}$ was subcloned into pT7HMT by incorporating 5' BamHI and 3' stop codon and NotI site using the pGEX4T2 construct containing ElpQ as template. Similarly, $ElpB_{19-378}$ was subcloned into pT7HMT by incorporating 5' SalI and 3' stop codon and NotI site using the pGEX4T2 construct containing ElpB as template. Subsequent expression and purification of BBK32-C, $ElpQ_{19-343}$, and $ElpB_{19-378}$ was completed as previously described (6). All proteins used in study were assessed for purity by SDS-PAGE prior to use in assays.

Quantitative ELISA to assess B. burgdorferi lipoprotein binding to purified human C1 components

One µg/well of purified human C1, C1q, C1r, or C1s proteins (Complement Technologies), or BSA (Sigma-Aldrich) as a negative control, were coated onto wells of an uncoated 96-well ELISA plate (Nunc Maxisorp) overnight at 4°C in coating buffer, as described above. The next day, plates were washed three times with PBST [10 mM disodium phosphate, 1.8 mM monopotassium phosphate, 137 mM sodium chloride, 2.7 mM potassium chloride, 0.05% (v/v)

Tween-20, pH 7.4] and blocked with 5% (w/v) nonfat dry milk in PBST. Plates were then washed, and 100 µl/well of four-fold dilutions of GST-tagged BBK32, ElpB, ElpQ, or BB0460 proteins, resulting in a range of concentrations from 1 µM to 240 pM, were added to the ELISA plate, which was then incubated for one hour at RT. Wells were washed and probed with 100 µl/well of a goat α-GST antibody (GE Healthcare Life Sciences, 27457701V) diluted 1:800 and incubated for one hour at RT. Wells were washed again and probed with 100 µl/well of a α-goat alkaline phosphatase conjugated antibody (Sigma Aldrich, A4187) diluted 1:2,000 and incubated for one hour at RT. Wells were washed a final time and the assay was developed using the SigmaFast pNpp reagent (Sigma Aldrich). OD405nm was read every minute for 15 minutes in a BioTek Synergy HT plate reader using Gen5 software. Substrate binding is expressed as the Vmean of ΔOD_{405nm}, calculated by determining the slope of the [OD_{405nm} vs. time] best fit line across the linear portion of the 15-minute kinetic assay. All experiments were repeated two to four times. K_D was quantified by a saturated binding parameter non-linear regression analysis performed using GraphPad Prism 6.0 software.

Surface plasmon resonance (SPR)

Binding of C1 and its sub-components to GST-ElpB and GST-ElpQ was performed at 25°C using a Biacore T200 (GE Healthcare) as previously described (6), with the following modifications. GST-ElpB and GST-ElpQ were amine coupled to the CMD200 (Xantec bioanalytics) at 10 µg/ml in 10 mM sodium acetate pH 4.0. Final immobilization densities shown in resonance units (RU) were 555.1 (GST-ElpB) and 451.1 RU (GST-ElpQ), and proenzyme studies were performed on 232.2 (GST-ElpB) and 485.7 (GST-ElpQ). C1s single cycle experiments had immobilization densities of 1181.3 (GST-ElpQ) and 1158.9 ($ElpQ_{19-343}$). HBS-T-Ca^{2+} (20 mM HEPES (pH 7.3), 140 mM NaCl, 0.005% (v/v) Tween 20, 5 mM $CaCl_2$) was used

as the running buffer and a flowrate of 30 µl min^{-1} was used in all experiments. All analytes were buffer exchanged into running buffer prior to experimentation.

Multicycle steady state analyses were performed as follows, C1 complex (Complement Technologies) was injected over flow cells in a two-fold concentration series: 0.59, 1.2, 2.3, 4.7, 9.4, 18.8, 37.5, 75, and 150 nM for 120 sec, followed by 180 sec dissociation. The same approach was used for C1r proenzyme, C1r enzyme, C1s proenzyme, and C1s enzyme (Complement Technologies), using a two-fold concentration series of 0.39, 0.78, 1.6, 3.1, 6.3, 13, 25, 50, 100, and 200 nM. Surfaces were then regenerated by injecting 2M NaCl for 60 sec 3 times consecutively, bringing the response to baseline. Alternatively, single cycle analysis was performed with a five-fold concentration series 0, 0.8, 4, 20, 100 nM with association times between each injection of 120 sec a final dissociation time of 600 sec. Kinetic analyses were performed on each sensorgram series using the Biacore T200 Evaluation Software 3.1 (GE Healthcare) and a 1:1 (Langmuir) binding model.

Gel-Based Inhibition of C1s-Mediated C2/C4 Cleavage Assay

To demonstrate inhibition of C1s mediated cleavage of C2 or C4 a 10 µL reaction in HBS-Ca^{2+} (10 mM HEPES (pH 7.3), 140 mM NaCl, 5 mM $CaCl_2$) was made by adding 6.25 nM C1s enzyme with twofold dilutions of ElpQ from 25,000 nM to 390 nM with subsequent addition of 1.25µL of C4 (1 mg/mL) (Complement Technologies). C2 assays were performed with 6.25 nM C1s enzyme with twofold dilutions of ElpQ or ElpB from 16,000 nM to 125 nM with subsequent addition of 1.25µL of C2 (0.5 mg/mL) (Complement Technologies). The reaction proceeded at 37° C for 1 hour and was stopped by the addition of 5 µL Laemmli buffer followed by boiling for 5 min. 10% SDS-PAGE gels were utilized with Coomassie staining. Gel imaging was completed on a ChemiDocTM XRS+ (Bio-Rad). Gels are representative of three independent experiments.

Gel-based C2/C4 inhibition assays were subjected to further quantitative analysis with Image Lab™ (Bio-Rad). Lanes and bands were manually selected and analyzed as follows. C4α' fragments were in lane normalized to C4b band and the background adjusted ratios are shown. C2b bands were in lane corrected for total C2 (C2 + C2b + C2a). 100% cleavage was constrained to C1s + C2/C4 control. A normalized four-parameter nonparametric response was analyzed in GraphPad v8.4.

Gel-Based Inhibition of C1r-Mediated Proenzyme C1s Cleavage Assay

Enzymatic inhibition assays were performed as previously described, with the following modifications (7). A 10 µL reaction in HBS-Ca^{2+} (10 mM HEPES (pH 7.3), 140 mM NaCl, 5 mM $CaCl_2$) was prepared by adding 1000 nM C1r to with twofold dilutions of $ElpQ_{19\text{-}343}$ from 25,000 nM to 390 nM and finally 1 µg proenzyme C1s. The reaction was incubated at 37º C for 1 hour and was stopped by the addition of 5 µL Laemmli buffer followed by boiling for 5 min. SDS-PAGE analysis was completed as in C2/C4 cleavage assay. Gel is representative of three independent experiments.

Inhibition of C3d and C5b-9 deposition by recombinant B. burgdorferi lipoproteins

96-well ELISA plates (Nunc Maxisorp) were coated with 300 ng human IgM (CP initiator) (Athens Research & Technology) in 100 µl/well of coating buffer (see above) overnight at 4°C. The following day, the plates were washed three times with PBS-T (see above) and were blocked with 200 µl of 1% (w/v) BSA (Sigma Aldrich) in PBS-T for one hour at RT. Normal human serum (Complement Technologies) was diluted to 2% (v/v) in CP reaction buffer [20 mM HEPES (pH 7.3), 140 mM sodium chloride, 150 µM calcium chloride, 500 µM magnesium chloride, 0.1% (w/v) gelatin]. GST-tagged ElpB, ElpQ, BBK32, or BB0460 proteins were serially diluted two-

fold and each dilution was mixed 1:1 with the above serum dilutions. 100 µl of each mixture was added to the complement initiator-treated wells. Plates were incubated in the presence of 5% CO2 for one hour at 37°C, followed by three washes with PBS-T. Following deposition, wells were blocked with 200 µl/well of 5% (w/v) nonfat dry milk in PBS-T for one hour at RT, followed by three washes with PBS-T. Wells were then probed with 100 µl/well of a mouse α-C3d (Abcam, ab17453) or α-C5b-9 (Santa Cruz, aE11) primary antibody diluted 1:500 and incubated for one hour at RT. Wells were washed again and probed with 100 µl/well of a α-mouse alkaline phosphatase-conjugated secondary antibody (Sigma Aldrich, A4187) diluted 1:2,000 and incubated for one hour at RT. Wells were washed a final time and developed using the SigmaFast pNpp reagent (Sigma Aldrich). OD_{405nm} readings were taken every minute for 15 minutes in a BioTek Synergy HT plate reader using Gen5 software. Substrate binding is expressed as the Vmean of ΔOD_{405nm}, which is calculated by determining the slope of the [OD_{405nm} vs. time] best fit line across the linear portion of the 15-minute kinetic assay. All experiments were repeated two to four times.

Inhibition of C4d deposition by recombinant B. burgdorferi lipoproteins

To show direct inhibition of classical pathway activation, an ELISA approach was used (6, 7). 3 ug mL^{-1} Human IgM (MP Biomedical), a classical pathway activator, in coating buffer (100 mM Na_2CO_3/$NaHCO_3$ pH 9.6 was immobilized overnight at room temperature in high-binding polypropylene microplates (Grenier bio-one). All subsequent steps were then washed three times, 100 µl volumes, with TBS-T (50mM Tris (pH 8.0), 150 mM NaCl, 0.05% (v/v) TritonX-100). Unbound regions of the plate were then blocked with PBS-T-BSA (137mM NaCl, 2.7 mM KCl, 10mM Na_2HPO_4,1.8 mM KH_2PO_4, 1% (w/v) bovine serum albumin, and 0.05% (v/v) Tween-20) for 1 h at 37°C. Classical pathway-mediated complement activation was then induced by adding

2% final pooled Normal Human Serum (NHS, Innovative Research) and a two-fold dilution series of GST-ElpB/GST-ElpQ/GST-BB0460 or untagged proteins BBK32-C/ElpQ$_{19\text{-}343}$, respectively, in CP Buffer (20 mM HEPES (pH 7.3), 0.1% (w/v) gelatin type A, 140 mM NaCl, 2 mM $CaCl_2$, 0.5 mM $MgCl_2$) with incubation at 37°C for 1 hr. A 1:300 dilution anti-C4 antibody (HYB 162-02) (Santa Cruz Biotechnology) in CP Buffer incubated at 37°C for 1 hour was used to detect complement activation. A 1:3000 dilution of goat anti-mouse HRP secondary antibody (Thermo Scientific) was then used at room temperature with light rocking for 1 hour. Activation of HRP conjugated antibody was detected by room temperature 1-step Ultra TMB ELISA (Thermo Scientific) for 10 min with rocking in the dark. The reaction was then stopped with the addition of 0.16 N sulfuric acid and the absorbance measured at 450 nM on an EnSight multimode plate reader (PerkinElmer). Data were in-column normalized using cells containing serum only or no serum with buffer addition were used as 100% and 0% signal, respectively. All experiments were performed in triplicate and IC_{50} values were determined using a variable four-parameter nonlinear regression analysis using GraphPad Prism 8.1.2.

Inhibition of erythrocyte hemolysis by recombinant B. burgdorferi lipoproteins

Inhibition of CP-mediated erythrocyte hemolysis by recombinant *B. burgdorferi* lipoproteins was assayed using a modified version of the previously described classical pathway hemolytic assay (6, 8). Normal human serum (Complement Technologies) was diluted to 2.3% (v/v) in CP reaction buffer [20 mM HEPES (pH 7.3), 140 mM sodium chloride, 150 μM calcium chloride, 500 μM magnesium chloride, 0.1% (w/v) gelatin]. GST-tagged ElpB, ElpQ, BBK32, or BB0460 proteins were serially diluted two-fold, 125 μl of each dilution was mixed with 125 μl of the diluted serum, and the mixtures were incubated at room temperature for one hour. During incubation, 5 ml of pre-opsonized sheep erythrocytes (Complement Technologies) were

centrifuged (400 × g, 3 minutes, 4°C) and washed twice in CP reaction buffer. After washing, erythrocytes were resuspended in 5 ml of CP buffer and 40 µl of the erythrocyte suspension were added to each of the incubated serum-protein mixtures. These reactions were incubated for one hour at room temperature, gently vortexing every 15 minutes to ensure that erythrocytes remained in suspension. Following incubation, samples were centrifuged (600 × g, 3 minutes, 4°C) and 200 µl of supernatant from each sample was collected and the OD405nm was measured in a BioTek Synergy HT plate reader using Gen5 software.

Inhibition of C1r and C1s enzyme activity by synthetic peptide cleavage

C1r enzyme and C1s enzyme assays were performed in HBS-Ca^{2+} (20 mM HEPES (pH7.3), 140 mM NaCl, 5 mM $CaCl_2$). C1r enzyme assays were completed by monitoring the autolytic activation of C1r proenzyme by adding GST-ElpB or GST-ElpQ, at a concentration of 25 µM, with 25 nM C1r proenzyme. Subsequent addition of 300 µM Z-Gly-Arg thiobenzyl (MP Biomedicals) and 100 µM 5,5′-dithiobis(2-nitrobenzoic acid) (DTNB) (TCI) just prior to measurement for a final 80 µl reaction volume (9). C1s enzyme assays were performed by adding GST-ElpB or GST-ElpQ, at a concentration of 25 µM, to 100 µM Z-L-Lys thiobenzyl and 100 µM DTNB. Just prior to measurement, 6.25 nM C1s enzyme was added for a final 80 µl reaction volume. Absorbance measurements were performed at 412 nM on a Versamax multimode plate reader (Molecular Devices) with plate reads occurring every 30 sec at 28° C for 3 hrs (C1r) and 37°C for 1 hr (C1s). Data were in-column normalized by including the C1r proenzyme or C1s enzyme with substrate as 100% signal, or just peptide and DTNB as 0%.

Classical pathway-mediated serum-killing assay

1×10^9 B31-e2 spirochetes expressing ElpB, ElpQ, BBK32, or BB0460 were harvested in late log phase by centrifugation (4,000 × g, 15 min). Supernatant was discarded and cell pellets were

washed three times in CP buffer [20 mM HEPES (pH 7.3), 140 mM NaCl, 150 μM $CaCl_2$, 500 μM $MgCl_2$, 0.1% gelatin]. After resuspending the pellet in 1 ml of CP buffer, the cells were split into three tubes containing 5×10^7 spirochetes each. 4 μg of an α-*B. burgdorferi* antibody (Abcam, ab20950) was added to two tubes, while its isotype control (Abcam, ab171870) was added to the third. Cell suspensions were incubated at RT for 1 hour, rocking.

Following incubation, the cells were pelleted, washed three times in CP buffer, and resuspended in 625 μl of CP buffer. From one of the α-Bb antibody tubes, as well as from the isotype control tube, 1×10^7 spirochetes (125 μl of cell suspension) was dispensed into tubes containing 125 μl of 40% normal human serum (in CP buffer), supplemented with 20 μg/ml lysozyme (Sigma, L6876), in triplicate. 125 μl of cell suspension from the other α-Bb antibody tube was dispensed into tubes containing 125 μl of 40% heat-inactivated human serum (in CP buffer), supplemented with 20 μg/ml lysozyme, in triplicate. Tubes were all mixed thoroughly by hand and incubated at 37°C, standing, for 4 hours.

Following the second incubation, the entire 250 μl of the serum-cell suspension mixture was transferred into a culture tube containing 2.25 ml of BSK-II, supplemented with the appropriate antibiotics. These cultures were allowed to grow out for 72 hours in normal growth conditions. After 72 hours of growth, the cultures were counted in duplicate by dark field microscopy. Samples were normalized to triplicate counts from the samples that were treated with control antibodies.

Supplemental Figures

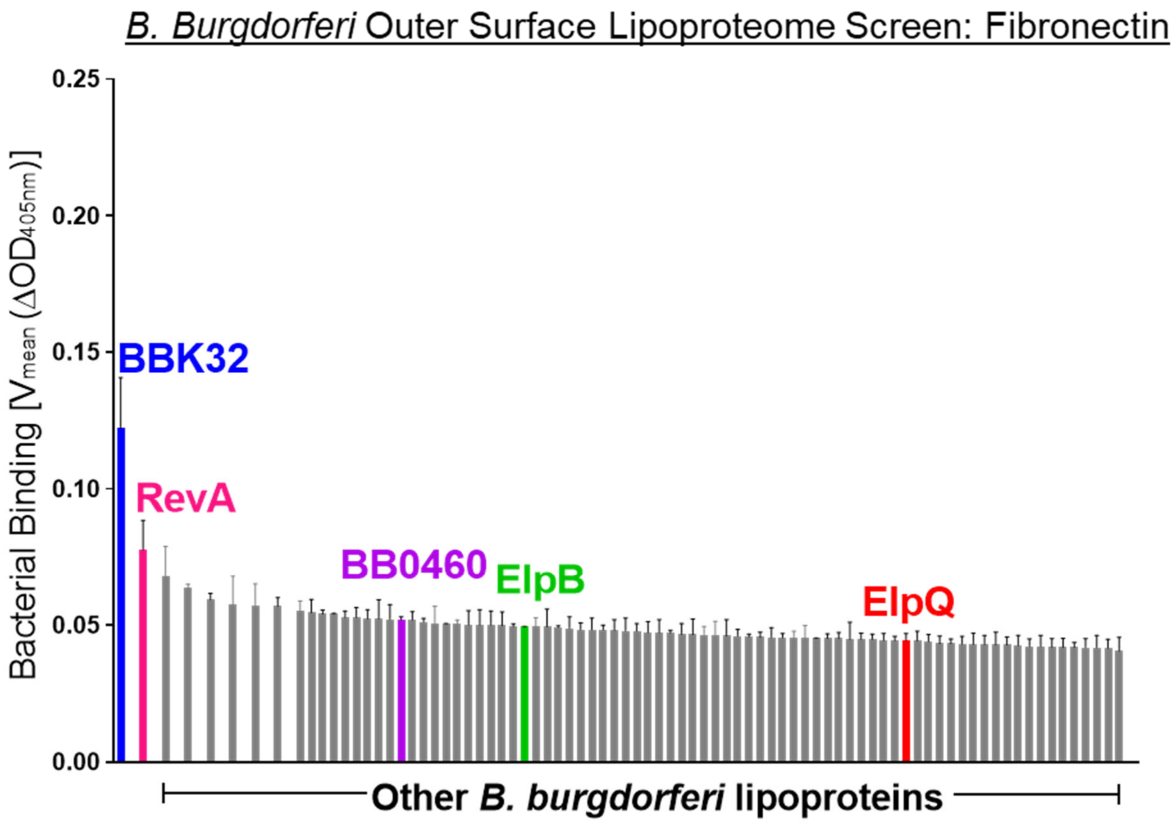

Figure S1. Validation of the Outer Surface Lipoproteome Screening Assay Using Human Fibronectin. The ability of *B. burgdorferi* strain B31-e2 overproducing lipoproteins to bind to human fibronectin immobilized in 96-well plates, reflected by the change in OD_{405nm} over time, is shown on the y-axis. The *B. burgdorferi* strain B31-e2 derivatives are sorted from left to right in order of efficiency of binding (see **Table S2**). Error bars indicate SEM. The two *B. burgdorferi* lipoproteins that have previously been identified as capable of binding directly to human fibronectin, BBK32 (blue) and RevA (pink), were also the two highest binding lipoproteins in the screen. Proteins used in this study for C1-binding are marked: ElpQ (red), ElpB (green), BB0460 (purple).

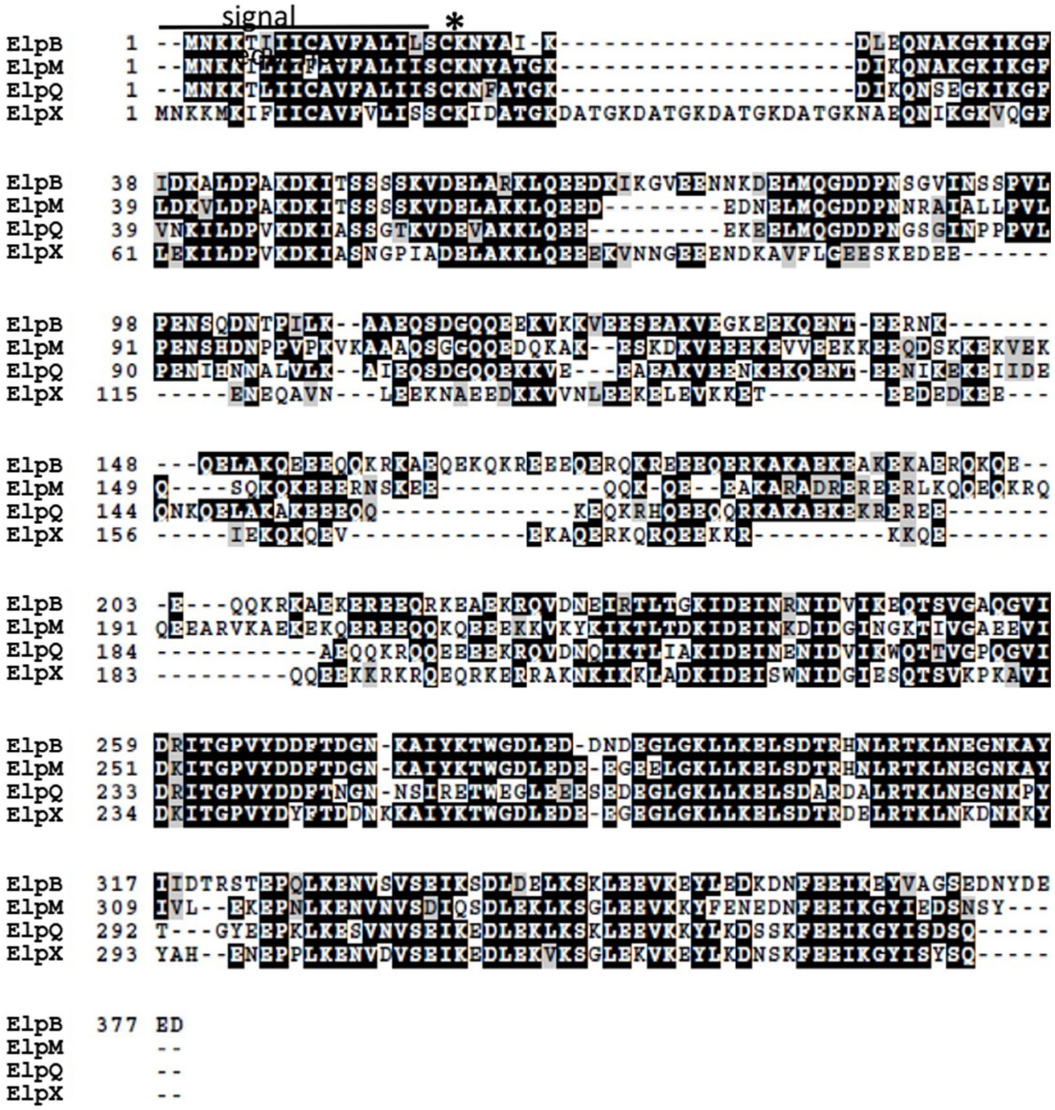

Figure S2. BOXSHADE alignment of Elp proteins. Asterisk indicates the site of lipoprotein acylation.

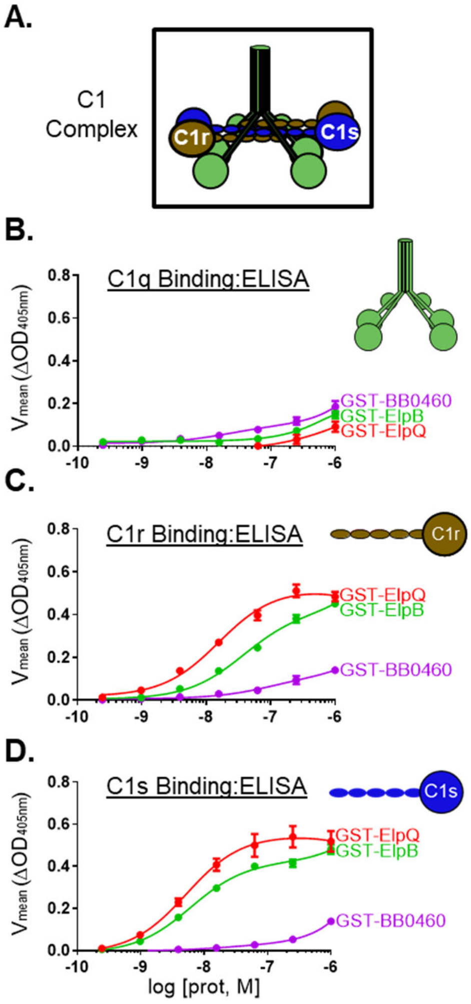

Figure S3. Binding of C1r and C1s by GST-ElpB and GST-ElpQ in ELISA-type Binding Assays. A) Model of C1 complex subcomponent structure. C1q (top, green), C1r (middle, brown), and C1s (bottom, blue). A four-fold dilution series of the indicated purified GST-fusion proteins were applied to wells coated with **B)** C1q, **C)** C1r enzyme, **D)** or C1s enzyme. Substrate binding is reported as the change in $OD_{405\ nm}$ over time. Error bars indicate SEM. Affinity analysis was performed on Prism GraphPad software, using a non-linear regression analysis fitting procedure and K_D values are presented in **Table 1**.

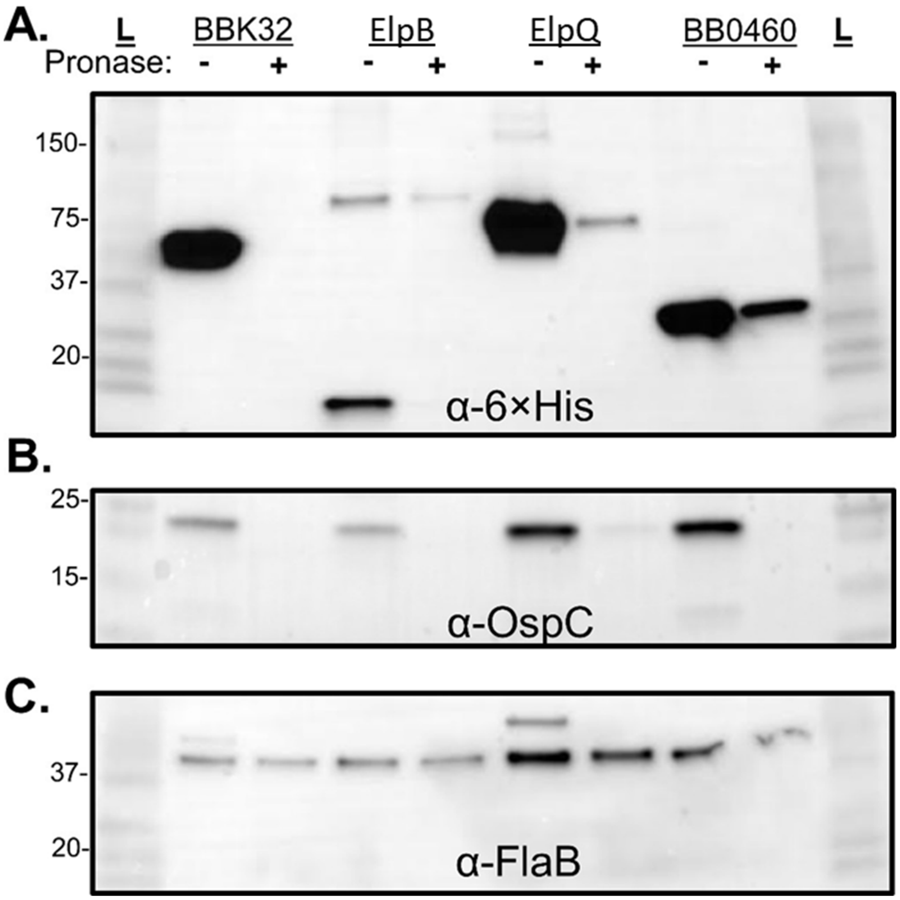

Figure S4. Validation of apparent molecular weight and localization of epitope-tagged BBK32, ElpB, ElpQ, and BB0460 expressed in *B. burgdorferi* strain B31-e2. 1×10^7 spirochetes +/-pronase treatment were lysed, resolved by SDS-PAGE, and transferred to a PVDF membrane. **A)** An α-6×His antibody, combined with HRP-conjugated Ni^{2+} beads, were used to determine protein levels in both treatment conditions. All observed bands correspond to the reported sizes of each protein. **B)** Antibodies to OspC or **C)** FlaB were used to assess pronase accessibility to the bacterial surface and periplasm, respectively.

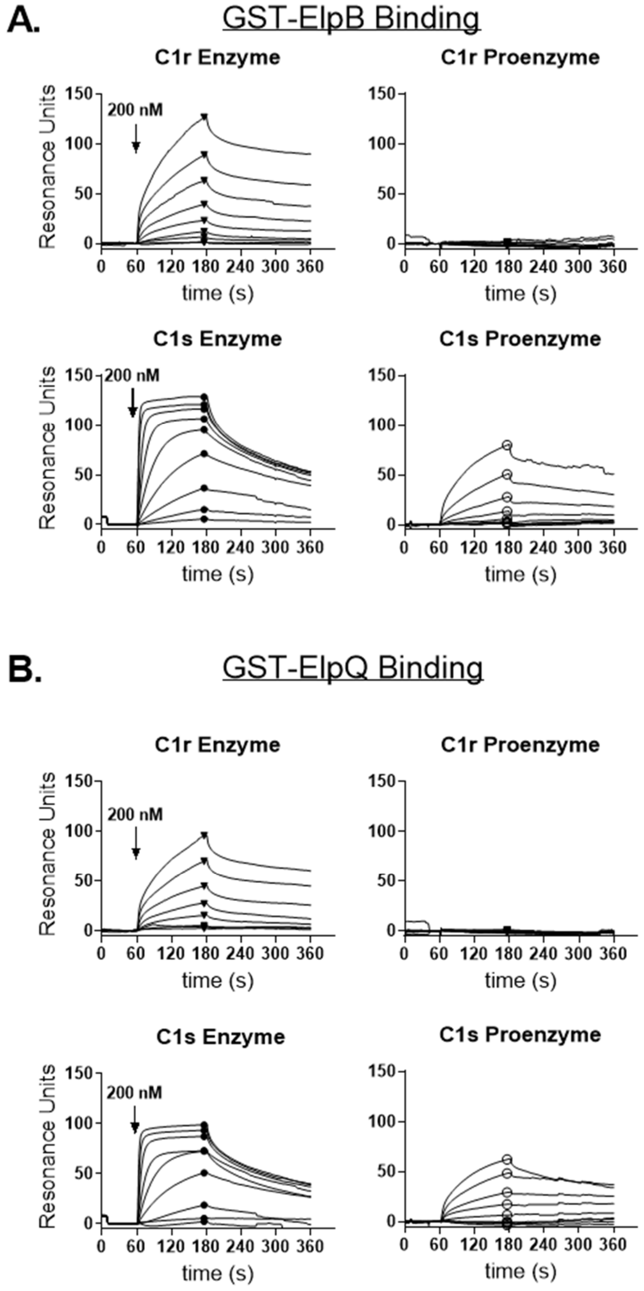

Figure S5. SPR sensorgrams for GST-ElpB and GST-ElpQ binding to C1r and C1s proenzyme and enzyme forms. A) GST-ElpB or **B)** GST-ElpQ raw sensorgrams are shown for C1r enzyme (top left), C1r proenzyme (top right), C1s enzyme (bottom left) and C1s proenzyme (bottom right). Symbols indicate the region of the sensorgram treated as steady-state signal for the fits shown in **Fig 2 C, D**.

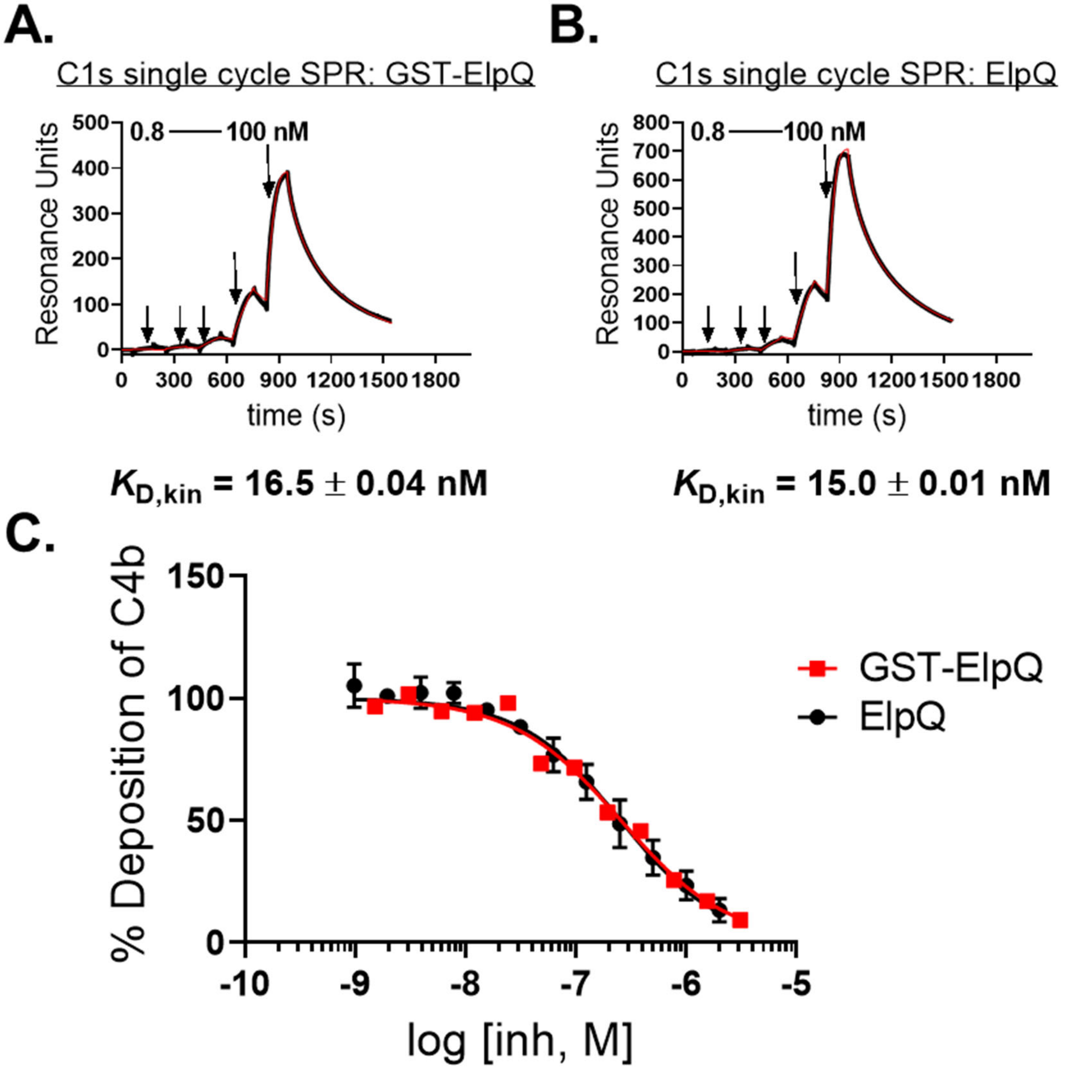

Figure S6. Validation of ElpQ Lacking a GST-affinity Tag. A-B) The interaction of C1s enzyme with GST-ElpQ and ElpQ lacking a GST tag was measured by SPR. A single cycle kinetic analysis was used for determination of K_D values (i.e. $K_{D,kin}$). A five-fold injection series (0, 0.8, 4, 20, and 100 nM) was repeated in triplicate. **C)** A classical pathway-specific ELISA-based complement assay using C4b detection was performed for ElpQ lacking a GST tag. The data for GST-ElpQ presented in **Fig 4A** are replotted here for comparisons sake. Each ElpQ concentration series was repeated in triplicate.

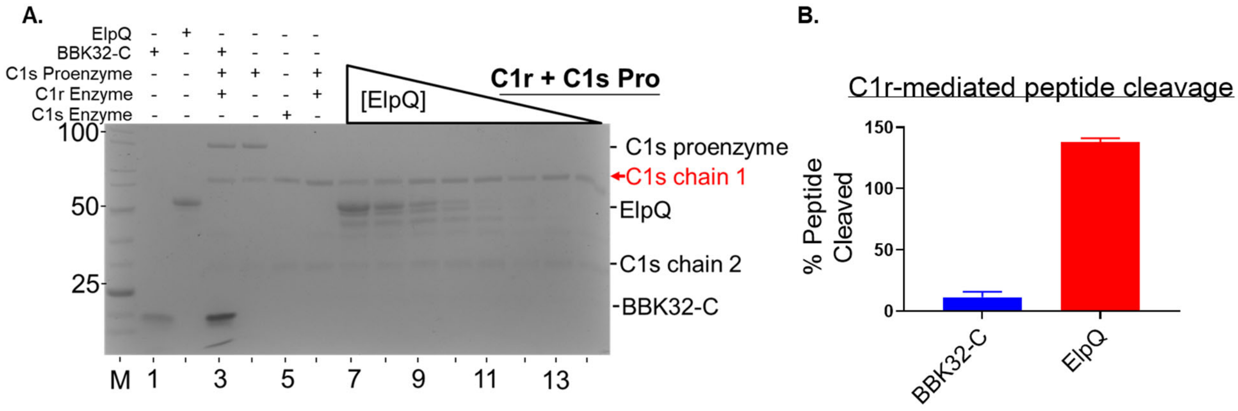

Figure S7. ElpQ does not inhibit C1r. A) The ability of ElpQ to block the C1r enzyme-mediated cleavage of C1s proenzyme was monitored by SDS-PAGE. Upon cleavage by C1r, C1s proenzyme is converted to two polypeptide chains of ~58 (chain 1) and ~28 kDa (chain 2). Lane M: Ladder, Lanes (1-6) various combinations of proteins in the assay denoting addition (+) or absence (-) of reagent. Lanes (7-14) concentrations of ElpQ up to 25 µM failed to prevent the cleavage of C1s proenzyme by C1r enzyme. BBK32-C was used as a positive control for C1r inhibition. **B)** C1r autolytic activation and enzymatic cleavage of active site analog Z-Gly-Arg-sBzl was assayed with DTNB (Ellman's reagent) in the presence of inhibitor (25 µM) at 25°C for 1hr. Absorbance was read at 412nm and signals were normalized to C1r proenzyme only wells. BBK32-C was used a positive control for inhibition of C1r proenzyme.

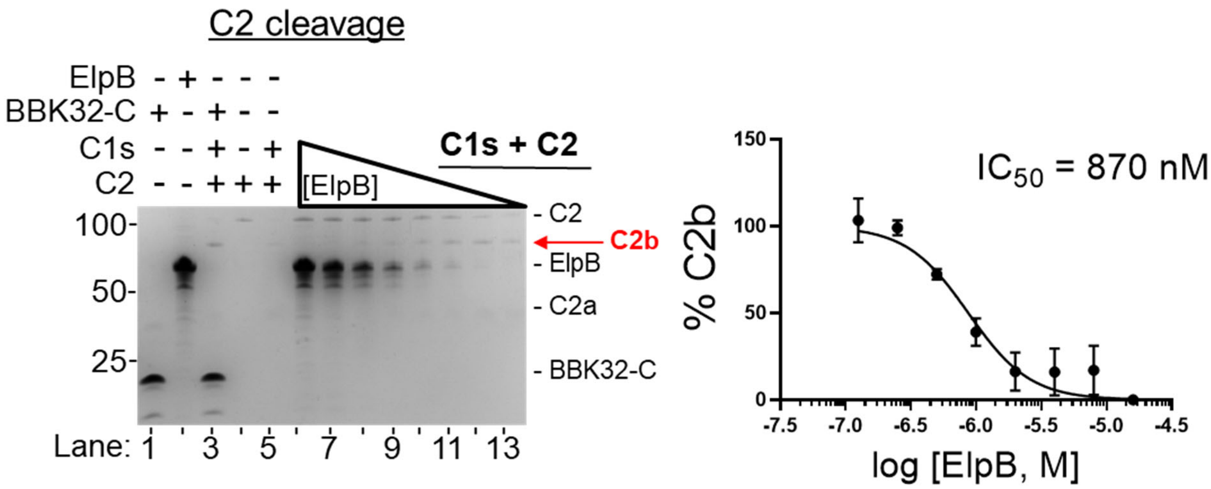

Figure S8. ElpB blocks C1s-mediated proteolytic cleavage of C2. Left: Proteolytic cleavage of C2 by C1s enzyme produces ~70kDa C2b and ~35kDa C2a after 1hr at 37°C. Lanes 1-5: C2b accumulation in the presence ("+") or absence ("-") of 16 μM ElpB, 25 μ M BBK32-C (non-inhibitory control), 6.25 nM C1s, and 685 nM C2. (Note that the amount of C1s loaded is below the level of detection by SDS-PAGE). Lanes 6-13: C2b accumulation in the presence of 6.25 nM C1s, 685 nM C2 and a two-fold dilution series (from 16 to 0.13 μM) of ElpB. A representative gel of triplicate experimentation is shown. Right: The fraction of C2b relative to total input C2 in the same lane determined by densitometry analysis data are normalized to C2 (lane 4) and C1s digested C2 (lane 5).

Table S1. Plasmids used in this study.

Name	Vector	Size (kb)	Insert	Size (bp)	Reference
pSC1116	pSC:LP	6.3	*bbk32*	1081	(1)
pSC1070	pSC:LP	6.3	*elpB* (*erpB*)[a]	1153	(1)
pSC1085	pSC:LP	6.3	*elpQ* (*erpQ*)[a]	1048	(1)
pSC1126	pSC:LP	6.3	*elpX* (*erpX*)[a]	1054	(1)
pSC1079	pSC:LP	6.3	*elpM* (*erpM*)[a]	1108	(1)
pSC1028	pSC:LP	6.3	*bb_0460*	730	(1)
pMP15	pGEX4T2	5	*bb_0460*	660	This study
pMP16	pGEX4T2	5	*bbk32*	1002	This study
pMP17	pGEX4T2	5	*elpQ* (*erpQ*)[a]	975	This study
pMP18	pGEX4T2	5	*elpB* (*erpB*)[a]	1080	This study
pElpQ	pT7HMT	5.4	*elpQ* (*erpQ*)[a]	975	This study
pElpB	pT7HMT	5.4	*elpB* (*erpB*)[a]	975	This study
pBBK32-C	pT7HMT	5.4	*bbk32 206-348*	426	(10)
pSC1037	pSC:LP	6.3	*bb_0158*	773	(1)
pSC1048	pSC:LP	6.3	*bb_0171*	604	(1)
pSC1047	pSC:LP	6.3	*bb_0213*	670	(1)
pSC1016	pSC:LP	6.3	*bb_0689*	484	(1)
pSC1039	pSC:LP	6.3	*bb_0758*	799	(1)
pSC1042	pSC:LP	6.3	*bb_0823*	388	(1)
pSC1059	pSC:LP	6.3	*bb_A04*	865	(1)
pSC1061	pSC:LP	6.3	*bb_A07*	505	(1)
pSC1068	pSC:LP	6.3	*bb_A14*	382	(1)
pSC1000	pSC:LP	6.3	*ospA*	958	(1)
pSC1009	pSC:LP	6.3	*ospB*	907	(1)
pSC1008	pSC:LP	6.3	*dbpA*	592	(1)
pSC1065	pSC:LP	6.3	*bb_A32*	211	(1)
pSC1054	pSC:LP	6.3	*bb_A33*	556	(1)
pSC1063	pSC:LP	6.3	*bb_A36*	658	(1)
pSC1053	pSC:LP	6.3	*bb_A57*	1261	(1)
pSC1057	pSC:LP	6.3	*bb_A59*	256	(1)
pSC1060	pSC:LP	6.3	*bb_A64*	940	(1)
pSC1064	pSC:LP	6.3	*bb_A65*	865	(1)
pSC1062	pSC:LP	6.3	*cspA*	772	(1)
pSC1058	pSC:LP	6.3	*bb_A69*	808	(1)
pSC1014	pSC:LP	6.3	*bb_B08*	646	(1)

pSC1002	pSC:LP	6.3	*ospC*	649	(1)
pSC1025	pSC:LP	6.3	*bb_B25*	529	(1)
pSC1087	pSC:LP	6.3	*bb_C10*	544	(1)
pSC1088	pSC:LP	6.3	*bb_D10*	595	(1)
pSC1091	pSC:LP	6.3	*bb_E04*	157	(1)
pSC1089	pSC:LP	6.3	*bb_E08*	163	(1)
pSC1090	pSC:LP	6.3	*bb_E31*	739	(1)
pSC1093	pSC:LP	6.3	*bb_F01*	1015	(1)
pSC1092	pSC:LP	6.3	*bb_F20*	310	(1)
pSC1095	pSC:LP	6.3	*bb_G01*	910	(1)
pSC1099	pSC:LP	6.3	*bb_H01*	211	(1)
pSC1098	pSC:LP	6.3	*cspZ*	727	(1)
pSC1096	pSC:LP	6.3	*bb_H18*	1132	(1)
pSC1097	pSC:LP	6.3	*bb_H32*	766	(1)
pSC1100	pSC:LP	6.3	*bb_H37*	955	(1)
pSC1107	pSC:LP	6.3	*bb_I14*	130	(1)
pSC1102	pSC:LP	6.3	*vraA*	1372	(1)
pSC1101	pSC:LP	6.3	*bb_I28*	589	(1)
pSC1108	pSC:LP	6.3	*bb_I29*	682	(1)
pSC1104	pSC:LP	6.3	*bb_I36*	853	(1)
pSC1105	pSC:LP	6.3	*bb_I38*	853	(1)
pSC1106	pSC:LP	6.3	*bb_I39*	883	(1)
pSC1103	pSC:LP	6.3	*bb_I42*	571	(1)
pSC1121	pSC:LP	6.3	*bb_J01*	202	(1)
pSC1011	pSC:LP	6.3	*ospD*	790	(1)
pSC1119	pSC:LP	6.3	*bb_J34*	1087	(1)
pSC1118	pSC:LP	6.3	*bb_J36*	1075	(1)
pSC1120	pSC:LP	6.3	*bb_J41*	883	(1)
pSC1117	pSC:LP	6.3	*bb_K01*	910	(1)
pSC1111	pSC:LP	6.3	*bb_K07*	769	(1)
pSC1110	pSC:LP	6.3	*bb_K12*	715	(1)
pSC1113	pSC:LP	6.3	*bb_K19*	652	(1)
pSC1112	pSC:LP	6.3	*bb_K48*	883	(1)
pSC1109	pSC:LP	6.3	*bb_K50*	1015	(1)
pSC1115	pSC:LP	6.3	*bb_K52*	865	(1)
pSC1114	pSC:LP	6.3	*bb_K53*	571	(1)
pSC1083	pSC:LP	6.3	*mlpH*	463	(1)
pSC1081	pSC:LP	6.3	*erpN*	541	(1)
pSC1076	pSC:LP	6.3	*mlpF*	466	(1)

pSC1075	pSC:LP	6.3	*erpK*	787	(1)
pSC1086	pSC:LP	6.3	*mlpI*	445	(1)
pSC1084	pSC:LP	6.3	*erpP*	568	(1)
pSC1071	pSC:LP	6.3	*revA*	490	(1)
pSC1069	pSC:LP	6.3	*mlpA*	463	(1)
pSC1010	pSC:LP	6.3	*erpA*	529	(1)
pSC1125	pSC:LP	6.3	*bb_Q03*	568	(1)
pSC1124	pSC:LP	6.3	*bb_Q05*	760	(1)
pSC1127	pSC:LP	6.3	*mlpJ*	628	(1)
pSC1128	pSC:LP	6.3	*bb_Q89*	211	(1)
pSC1074	pSC:LP	6.3	*mlpD*	439	(1)
pSC1073	pSC:LP	6.3	*bb_R40*	121	(1)
pSC1012	pSC:LP	6.3	*bb_R42*	691	(1)
pSC1072	pSC:LP	6.3	*mlpC*	463	(1)
pSC1013	pSC:LP	6.3	*erpG*	607	(1)

[a]Previous *erp* terminology is given in parentheses. (See text.)

Table S2. Fn- and C1-binding by *Bb* strain B31-e2 that ectopically produce *Bb* surface lipoproteins

Gene locus	Location	Gene name	Fn-binding[a]	C1-binding[b]
bb_0460[c]	chromosome		0.0521	0.0488
bb_0158	chromosome		0.0519	0.0576
bb_0171	chromosome		0.0501	0.0553
bb_0213	chromosome		0.0501	0.0522
bb_0689	chromosome		0.0495	0.0511
bb_0758	chromosome		0.0501	0.0486
bb_0823	chromosome		0.0521	0.0484
bb_A04	lp54		0.0550	0.0504
bb_A07	lp54	*chpAI*	0.0546	0.0492
bb_A14	lp54		0.0529	0.0521
bb_A15	lp54	*ospA*	0.0570	0.0533
bb_A16	lp54	*ospB*	0.0541	0.0510
bb_A24	lp54	*dbpA*	0.0594	0.0504
bb_A32	lp54		0.0499	0.0520
bb_A33	lp54		0.0460	0.0481
bb_A36	lp54		0.0429	0.0469
bb_A57	lp54		0.0456	0.0454
bb_A59	lp54		0.0433	0.0441
bb_A64	lp54		0.0449	0.0450
bb_A65	lp54		0.0464	0.0443
bb_A68	lp54	*cspA*	0.0455	0.0422
bb_A69	lp54		0.0452	0.0483
bb_B08	cp26		0.0529	0.0500
bb_B19	cp26	*ospC*	0.0493	0.0490
bb_B25	cp26		0.0523	0.0518
bb_C10	cp9	*revB*	0.0449	0.0478
bb_D10	lp17		0.0440	0.0451
bb_E04	lp25		0.0416	0.0415
bb_E08	lp25		0.0407	0.0405
bb_E31	lp25		0.0479	0.0562
bb_F01	lp28-1	*erpD*	0.0420	0.0427
bb_F20	lp28-1		0.0419	0.0399

bb_G01	lp28-2		0.0454	0.0428
bb_H01	lp28-3		0.0453	0.0423
bb_H06	lp28-3	*cspZ*	0.0444	0.0427
bb_H18	lp28-3		0.0505	0.0459
bb_H32	lp28-3		0.0506	0.0506
bb_H37	lp28-3		0.0507	0.0508
bb_I14	lp28-4		0.0420	0.0459
bb_I16	lp28-4	*vraA*	0.0418	0.0407
bb_I28	lp28-4		0.0463	0.0425
bb_I29	lp28-4		0.0512	0.0491
bb_I36	lp28-4		0.0496	0.0476
bb_I38	lp28-4		0.0456	0.0428
bb_I39	lp28-4		0.0575	0.0510
bb_I42	lp28-4		0.0443	0.0403
bb_J01	lp38		0.0430	0.0408
bb_J09	lp38	*ospD*	0.0428	0.0402
bb_J34	lp38		0.0420	0.0408
bb_J36	lp38		0.0680	0.0552
bb_J41	lp38		0.0573	0.0476
bb_K01	lp36		0.0495	0.0432
bb_K07	lp36		0.0472	0.0425
bb_K12	lp36		0.0433	0.0412
bb_K19	lp36		0.0431	0.0431
bb_K32	lp36	*bbk32*	0.1221	0.1425
bb_K48	lp36		0.0454	0.0415
bb_K50	lp36		0.0638	0.0485
bb_K52	lp36		0.0426	0.0421
bb_K53	lp36		0.0430	0.0436
bb_L28	cp32-8	*mlpH*	0.0478	0.0468
bb_L39	cp32-8	*erpN*	0.0489	0.0481
bb_M28	cp32-6	*mlpF*	0.0445	0.0479
bb_M38	cp32-6	*erpK*	0.0422	0.0431
bb_N28	cp32-9	*mlpI*	0.0449	0.0438
bb_N38	cp32-9	*erpP*	0.0423	0.0438
bb_N39	cp32-9	*elpQ* (*erpQ*)[d]	0.0444	0.2230
bb_P27	cp32-1	*revA*	0.0777	0.0444
bb_P28	cp32-1	*mlpA*	0.0453	0.0440

bb_P38	cp32-1	*erpA*	0.0458	0.0439
bb_P39	cp32-1	*elpB* (*erpB*)[d]	0.0495	0.2064
bb_Q03	lp56		0.0484	0.0479
bb_Q05	lp56		0.0541	0.0515
bb_Q35	lp56	*mlpJ*	0.0523	0.0540
bb_Q47	lp56	*elpX* (*erpX*)[d]	0.0478	0.0703
bb_Q89	lp56		0.0468	0.0487
bb_R28	cp32-4	*mlpD*	0.0474	0.0495
bb_R40	cp32-4	*erpH*	0.0461	0.0496
bb_R42	cp32-4	*erpY*	0.0482	0.0483
bb_S30	cp32-3	*mlpC*	0.0466	0.0514
bb_S41	cp32-3	*erpG*	0.0484	0.0479

[a]Expressed as change in OD_{405nm} over time in ELISA-based quantitation of binding to immobilized Fn, as described in Fig S1 legend and Methods.

[b]Expressed as change in OD_{405nm} over time in ELISA-based quantitation of binding to immobilized C1, as described in Fig 1 legend and Methods.

[c]Encodes a lipoprotein previously suggested to be largely periplasmic (1) and was used as a negative control.

[d]Previous *erp* terminology is given in parentheses (see text.)

Table S3. ElpB and ElpQ do not enhance spirochetal survival when serum is heat-treated or in the absence of specific Ab[a].

Lipoprotein produced	Culture density (bacteria/ml)	
	Control Ab + serum[b]	Anti-Bb Ab + Heat-Tx'ed serum[c]
BB0460	6.02 (± 0.48) × 10^7	3.98 (± 0.70) × 10^7
ElpB	7.20 (± 0.32) × 10^7	7.90 (± 0.18) × 10^7
ElpQ	1.63 (± 0.28) × 10^7	6.17 (± 0.69) × 10^7
BBK32	1.09 (± 0.04) × 10^8	8.72 (± 0.87) × 10^7

[a]Representative of two independent experiments.

[b]Mean (± SEM) of triplicate determination of culture density after 5 × 10^7 B31-e2 spirochetes expressing the indicated protein were treated with control antibody, human serum, and lysozyme, then cultured for 72 hours in 2.5 ml BSK-II (see Materials and Methods).

[c]Mean (± SEM) of triplicate determination of culture density after 5 × 10^7 B31-e2 spirochetes expressing the indicated protein were treated with anti-Bb antibody, heat-treated serum, and lysozyme, then cultured for 72 hours in 2.5 ml BSK- II (see Materials and Methods).

Table S4. Degree of identity and similarity among *B. burgdorferi* B31 Elp proteins.

	ElpB	ElpM	ElpO	ElpQ	ElpX
**ElpB**		75.7%	100%	73.1%	59.4%
**ElpM**	58.9%		75.7%	70.1%	59.0%
**ElpO**	100%	58.9%		73.1%	59.4%
**ElpQ**	58.0%	54.9%	58.0%		63.5%
**ElpX**	43.6%	45.4%	43.6%	47.4%	

%Similarity

%Identity

Table S5. Primers used in this study.

Name	Primer Sequence	Notes
prMP164	gctagGGATCCTCTAAATCAGTCTCAAGTA	Forward primer to amplify the *bb_0460* gene
prMP165	gctagCCCGGGAGACTGAGAGTG	Reverse primer to amplify the *bb_0460* gene
prMP177	gctagGGATCCGATTTATTCATAAGATATG	Forward primer to amplify the *bbk32* gene
prMP178	gctagCCCGGGGTACCAAACGC	Reverse primer to amplify the *bbk32* gene
prMP179	gctagGGATCCAAGAATTTTGCAACT	Forward primer to amplify the *elpQ* gene
prMP180	gctagCCCGGGCTGACTGTCA	Reverse primer to amplify the *elpQ* gene
prMP181	gctagGAATTCCTAAGAATTATGCAATTAAAG	Forward primer to amplify the *elpB* gene
prMP182	gctagCCCGGGATCTTCTTCATCATA	Reverse primer to amplify the *elpB* gene
$ElpQ_{19-343}F$	ggatccAAGAATTTTGCAACTGGTAA	Forward primer to amplify the *elpQ* gene for pT7HMT
$ElpQ_{19-343}R$	gcggccgcTTACTGACTGTCACTGATGTATC	Reverse primer to amplify the *elpQ* gene for pT7HMT
$ElpB_{19-378}F$	gtcgacTAAGAATTATGCAATTAAAGATTTAGAACAAAATG	Forward primer to amplify the *elpB* gene for pT7HMT
$ElpB_{19-378}R$	gcggccgcTTAATCTTCTTCATCATAATTATCCT	Reverse primer to amplify the *elp*B gene for pT7HMT

Supplemental References

1. A. S. Dowdell, *et al.*, Comprehensive spatial analysis of the *Borrelia burgdorferi* lipoproteome reveals a compartmentalization bias toward the bacterial surface. *J. Bacteriol.* **199**, E00658 (2017).

2. J. R. Fischer, N. Parveen, L. Magoun, J. M. Leong, Decorin-binding proteins A and B confer distinct mammalian cell type-specific attachment by Borrelia burgdorferi, the Lyme disease spirochete. *Proc. Natl. Acad. Sci. U. S. A.* **100**, 7307–7312 (2003).

3. W. S. Probert, B. J. Johnson, Identification of a 47 kDa fibronectin-binding protein expressed by *Borrelia burgdorferi* isolate B31. *Mol. Microbiol.* **30**, 1003–1015 (1998).

4. Y. Wu, Q. Li, X. Z. Chen, Detecting protein-protein interactions by far western blotting. *Nat. Protoc.* **2**, 3278–3284 (2007).

5. V. M. Benoit, J. R. Fischer, Y.-P. Lin, N. Parveen, J. M. Leong, Allelic Variation of the Lyme Disease Spirochete Adhesin DbpA Influences Spirochetal Binding to Decorin, Dermatan Sulfate, and Mammalian Cells. *Infect. Immun.* **79**, 3501–3509 (2011).

6. B. L. Garcia, H. Zhi, B. Wager, M. Höök, J. T. Skare, *Borrelia burgdorferi* BBK32 Inhibits the Classical Pathway by Blocking Activation of the C1 Complement Complex. *PLoS Pathog.* **12**, e1005404 (2016).

7. A. Roos, *et al.*, Functional characterization of the lectin pathway of complement in human serum. *Mol. Immunol.* **39**, 655–668 (2003).

8. U. R. Nilsson, B. Nilsson, Simplified assays of hemolytic activity of the classical and alternative complement pathways. *J. Immunol. Methods* **72**, 49–59 (1984).

9. J. J. Kardos, *et al.*, The Role of the Individual Domains in the Structure and Function of the Catalytic Region of a Modular Serine Protease, C1r. *J. Immunol.* **167**, 5202–5208 (2001).

10. J. Xie, *et al.*, Structural determination of the complement inhibitory domain of borrelia burgdorferi BBK32 provides insight into classical pathway complement evasion by lyme disease spirochetes. *PLoS Pathog.* **15**, e1007659 (2019).
